# Arcuate Angiotensin II Increases Arterial Pressure via Coordinated Increases in Sympathetic Nerve Activity and Vasopressin Secretion

**DOI:** 10.1523/ENEURO.0404-21.2021

**Published:** 2022-01-19

**Authors:** Zhigang Shi, Daniel S. Stornetta, Ruth L. Stornetta, Virginia L. Brooks

**Affiliations:** 1 Department of Chemical Physiology and Biochemistry, Oregon Health & Science University, Portland, OR 97239; 2 Department of Pharmacology, University of Virginia, Charlottesville, VA 22908

**Keywords:** angiotensin II, arcuate nucleus, autonomic nervous system, neuropeptide Y, POMC, vasopressin

## Abstract

The arcuate nucleus (ArcN) is an integrative hub for the regulation of energy balance, reproduction, and arterial pressure (AP), all of which are influenced by Angiotensin II (AngII); however, the cellular mechanisms and downstream neurocircuitry are unclear. Here, we show that ArcN AngII increases AP in female rats via two phases, both of which are mediated via activation of AngII type 1 receptors (AT1aRs): initial vasopressin-induced vasoconstriction, followed by slowly developing increases in sympathetic nerve activity (SNA) and heart rate (HR). In male rats, ArcN AngII evoked a similarly slow increase in SNA, but the initial pressor response was variable. In females, the effects of ArcN AngII varied during the estrous cycle, with significant increases in SNA, HR, and AP occurring during diestrus and estrus, but only increased AP during proestrus. Pregnancy markedly increased the expression of AT1aR in the ArcN with parallel substantial AngII-induced increases in SNA and MAP. In both sexes, the sympathoexcitation relied on suppression of tonic ArcN sympathoinhibitory neuropeptide Y (NPY) inputs, and activation of proopiomelanocortin (POMC) projections, to the paraventricular nucleus (PVN). Few or no NPY or POMC neurons expressed the AT1aR, suggesting that AngII increases AP and SNA at least in part indirectly via local interneurons, which express tyrosine hydroxylase (TH) and VGat (i.e., GABAergic). ArcN TH neurons release GABA locally, and central AT1aR and TH neurons mediate stress responses; therefore, we propose that TH AT1aR neurons are well situated to locally coordinate the regulation of multiple modalities within the ArcN in response to stress.

## Visual Abstract

**Figure:**
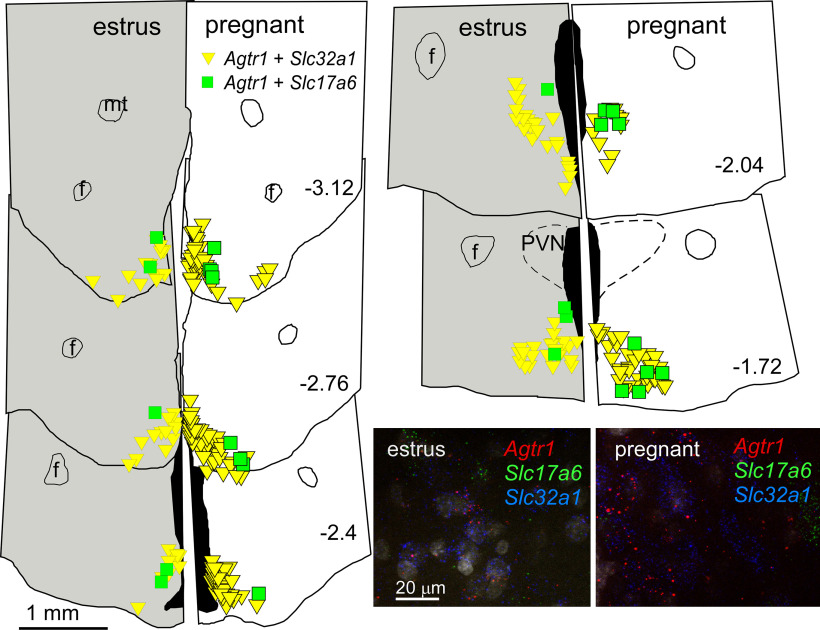

## Significance Statement

The arcuate nucleus (ArcN) is an integrative hub for the regulation of energy balance, reproduction, and arterial pressure (AP), all of which are influenced by Angiotensin II (AngII). Here, we show that ArcN AngII activates AngII type 1 receptors (AT1aRs) to increase AP in male and female rats by slowly increasing sympathetic nerve activity (SNA). In females, ArcN AngII also evoked an initial pressor response mediated by vasopressin-induced vasoconstriction. Pregnant and estrus females responded more than males, in association with higher ArcN AT1aR expression. AT1aRs were identified in ArcN interneurons that express tyrosine hydroxylase (TH) and GABA. Since brain AT1aR and TH mediate stress responses, ArcN AT1aR TH neurons are well situated to locally coordinate autonomic, hormonal, and behavioral responses to stress.

## Introduction

The hypothalamic arcuate nucleus (ArcN) is a well-established integrative hub for the regulation of energy balance and reproduction. The ArcN has also been identified as a site important in autonomic control of the cardiovascular system (Sapru, 2013). For example, the metabolic hormones, insulin and leptin, each act in the ArcN to increase arterial pressure (AP) and sympathetic nerve activity (SNA) to several organs, including skeletal muscle, the splanchnic circulation, and the kidneys (Cassaglia et al., 2011; Harlan and Rahmouni, 2013). Like insulin and leptin, ArcN Angiotensin II (AngII) increases AP (Arakawa et al., 2011). ArcN AngII also influences reproduction and energy balance (Mehay et al., 2021), implicating ArcN AngII as a candidate integrative neuropeptide. However, the mechanisms by which AngII increases AP, the cellular mechanisms of integration, and downstream neurocircuitry are unknown.

Two major ArcN cell types that influence AP and SNA are inhibitory neuropeptide Y (NPY) neurons and excitatory proopiomelanocortin (POMC) neurons, which release α-melanocyte stimulating hormone (α-MSH). Indeed, both leptin (Shi et al., 2015b) and insulin (Ward et al., 2011; Cassaglia et al., 2016) increase SNA via suppression of tonically sympathoinhibitory NPY neurons and activation of sympathoexcitatory POMC neurons. At least in mice, AngII type 1 receptors (AT1aRs) are highly expressed in ArcN AgRP neurons (Claflin et al., 2017), almost all of which also express NPY (Broberger et al., 1998). AgRP/NPY neurons inhibit SNA via release of NPY in two hypothalamic sites: the paraventricular nucleus (PVN) and dorsal medial hypothalamus (DMH; Shi et al., 2017). Therefore, we first tested whether ArcN AngII increases SNA in part by inhibiting NPY neurons that project to the PVN or the DMH. Second, because suppression of tonic PVN NPY sympathoinhibition can unveil the sympathoexcitatory effects of α-MSH at melanocortin type 3 or 4 receptors (MC3/4R; Cassaglia et al., 2014; Shi et al., 2015b), we also determined whether α-MSH contributes to the sympathoexcitatory effects of ArcN AngII.

Cardiovascular diseases that are sexually dimorphic, like hypertension, exhibit stark sex differences in a dependence on the renin-angiotensin system (RAS; for review, see Xue et al., 2013; Brooks et al., 2015; Ramirez and Sullivan, 2018). AT1aR are expressed more highly in the ArcN in females than in males, with the greatest levels observed during estrus/diestrus compared with proestrus (Seltzer et al., 1993; Jöhren et al., 1997). However, whether ArcN AngII also elicits cardiovascular and autonomic effects in females has not been previously investigated. Therefore, we next tested whether ArcN AngII increases SNA and AP in female rats and whether the response varies during the reproductive cycle. Second, as in males, we tested whether the sympathoexcitatory response relies on inverse changes in the activity of ArcN NPY and POMC neurons that project to the PVN.

Pregnancy increases SNA, likely due in part to the central actions of AngII (Brooks et al., 2020); however, the brain sites are unknown. The ArcN supports increased SNA during pregnancy (Shi et al., 2015a), but neither leptin nor insulin are involved (Shi et al., 2019b). Thus, the hormonal mediator has not been identified. Therefore, to begin to test the hypothesis that AngII acts in the ArcN to increase SNA during normal pregnancy, we determined whether ArcN AngII is sympathoexcitatory in late pregnant rats and whether this is associated with increased ArcN AT1AR expression. Aberrant activity of the RAS contributes to the often-fatal hypertensive disorder, preeclampsia, which increases SNA even more (Brooks et al., 2020). Thus, this information from normal pregnancy will also provide a basis for studies to test whether central actions of the RAS contribute to the excessive sympathoexcitation observed in females with pregnancy-induced hypertensive disorders.

Lastly, while the AT1aR was frequently found in NPY neurons in mice (Claflin et al., 2017), whether the same is true in rats is unknown. Therefore, we systematically explored the expression pattern and cellular phenotypes of the AT1aR in the ArcN of male rats and of female rats in various reproductive stages using fluorescent *in situ* hybridization (FISH).

## Materials and Methods

Experiments were performed using male and female Sprague Dawley rats (13–17 weeks, Charles River Laboratories, Inc). All the rats were acclimated for more than or equal to one week before experimentation in a room with a 12/12 h light/dark cycle, with food (LabDiet 5001) and water provided *ad libitum*. Rats were generally housed in pairs. Vaginal epithelial cytology was examined daily to establish the 4- to 5-d estrous cycle. Rats were usually impregnated by housing with a male, and the presence of vaginal sperm was designated pregnancy day 0 (P0). Alternatively, timed pregnant rats were obtained from Charles River Laboratories, Inc. Pregnant rats were housed singly, until the experiment on pregnancy day 20 (P20). All procedures were conducted in accordance with the National Institutes of Health *Guide for the Care and Use of Laboratory Animals* and approved by the Institutional (Oregon Health & Science University or University of Virginia) Animal Care and Use Committee.

### Experiments in anesthetized rats

#### Surgical preparation

Anesthesia was induced and maintained with 2–5% isoflurane in 100% oxygen. Body temperature was maintained at 37 ± 1°C using a rectal thermistor and heating pad. A tracheal tube, a femoral arterial catheter, and two venous catheters were placed for artificial ventilation, the measurement of mean AP (MAP), and drug infusions, respectively. The lumbar sympathetic nerve was located after a midline abdominal incision, and the splanchnic nerve was exposed after a flank incision. Bipolar stainless-steel electrodes were positioned and secured around the nerves using lightweight silicone material (Kwik-Sil, WPI, Inc). The rat was then placed in a stereotaxic instrument (David Kopf Instruments), and, following a midline incision on the top of the skull, a hole was burred near the midline to allow for ArcN, PVN, or DMH nanoinjections. After completion of surgery, isoflurane anesthesia was slowly withdrawn over 30 min, and a continuous intravenous (iv) infusion of α-chloralose was begun and continued for the duration of the experiment (50 mg/kg loading dose over 30 min; 25 mg/kg/h maintenance dose; Sigma-Aldrich). Pregnant rats received an α-chloralose dose equivalent to a virgin rat at a similar age. Throughout the experiment, the rats were continuously artificially ventilated with 100% oxygen, and respiratory rate and tidal volume were adjusted to maintain expired CO_2_ at 3.5–4.5%. Anesthetic depth was regularly confirmed by the lack of a pressor response to a foot or tail pinch; if necessary, additional α-chloralose was administered iv. After completion of surgery and the α-chloralose loading dose, rats were allowed to stabilize for ≥60 min before experimentation.

#### Experimental protocols

Hypothalamic nanoinjections were usually conducted over ∼5–10 s bilaterally (with ∼2  min between sides) using a pressure injection system (Pressure System IIe, Toohey Company) and single‐barreled glass micropipettes. All drugs were dissolved in artificial CSF (aCSF) containing the following: 128 mmol/l NaCl, 2.6 mmol/l KCl, 1.3 mmol/l CaCl_2_, 0.9 mmol/l MgCl_2_, 20 mmol/l NaHCO_3_, and 1.3 mmol/l Na_2_HPO_4_; pH was corrected to 7.4, and the aCSF was filtered before use. Briefly, with a flat skull and using the bregma and the dorsal surface of the dura as zero, the micropipette (20- to 40-μm tip o.d.) was positioned using the following coordinates: ArcN. 3.3–3.6 mm caudal, 0.3 mm lateral, and 9.8–10.2 mm ventral; PVN. 1.8–2.1 mm caudal, 0.5 mm lateral and 7.4–7.8 mm ventral; DMH. 3.2–3.3 mm caudal, 0.5 mm lateral, 8.5–8.7 mm ventral.

Experimental protocols were then performed to answer the following questions. (1) Does ArcN AngII increase SNA in males and females, and does the response vary during the estrous cycle or with pregnancy? After collecting baseline data, 30 nl of AngII (1 mm/l, Tocris) or aCSF was injected bilaterally into the ArcN and recordings continued for 90 min. (2) Is the SNA response mediated by ArcN AT1aR? A total of 60 nl of candesartan (0.5 mmol/l, Tocris Bioscience) or aCSF was injected bilaterally into the ArcN, and 10–15 min later, 30 nl of AngII [1 mmol/l (Arakawa et al., 2011), Tocris] or aCSF was injected bilaterally into the ArcN. In females, two estrous, one diestrous, and one pregnant rat were tested. (3) What is the role of NPY projections to the PVN and DMH? Leptin and insulin increase SNA by simultaneously decreasing NPY inhibitory actions at Y1 receptors (Y1Rs), and increasing α-MSH excitatory actions at MC3/4R, in the PVN (Ward et al., 2011; Shi et al., 2015b; Cassaglia et al., 2016). Therefore, prior blockade of NPY Y1R would not be expected to prevent the effects of ArcN AngII, even if NPY were involved, since α-MSH could still act unimpeded. Therefore, to test whether ArcN suppresses NPY inputs to the PVN, we instead first bilaterally injected AngII (1 mmol/l) into the ArcN. One hour later, we determined whether the sympathoexcitatory effects of the selective NPY Y1R antagonist, BIBO3304 [1 mmol/l (Cassaglia et al., 2014), Tocris], injected bilaterally into the PVN or DMH (males only), were abolished. In separate groups of animals, as a control, aCSF was injected instead of BIBO3304. Recordings were continued for another 30 min. (4) Do PVN MC3/4R mediate the sympathoexcitatory effect of ArcN AngII? (A) AngII (1 mmol/l) was injected bilaterally into the ArcN. At least 90 min later, the MC3/4R antagonist, SHU9119 (60 nl of 0.5 mmol/l in aCSF with 10% DMSO, Tocris), was injected into the PVN and variables were monitored for another 20–30 min. (B). In male rats, SHU9119 (60 nl of 0.5 mmol/l in aCSF with 10% DMSO, Tocris) or aCSF with 10% DMSO was injected bilaterally into the PVN, and 10–15 min later, AngII (1 mmol/l) was injected into the ArcN. (5) Does vasopressin contribute to the pressor response induced by ArcN AngII (females only)? After stabilization, the V1a vasopressin receptor antagonist (V1ax; Manning Compound, V2255, Sigma-Aldrich; 5 μg in 0.1-ml saline) or saline was given iv. 15 min later, aCSF or AngII was injected blilaterally into the ArcN.

At the end of each experiment, ∼60 nl of fluorescent polystyrene microbeads (FluoSpheres, F8803, 1:200; Invitrogen) were administered using the same pipette and coordinates to verify the injection sites using a standard anatomic atlas (Paxinos and Watson, 2007). Rats were then euthanized via iv administration of a barbiturate (Euthasol; Virbac AH).

#### Data analysis

Throughout the experiment, pulsatile AP, MAP, and heart rate (HR) were continuously collected using a Biopac MP100 data acquisition and analysis system (Biopac Systems), sampling at 2000 Hz. SNA was band‐pass filtered (100–3000 Hz) and amplified (×10,000). After data collection, postmortem SNA was quantified and subtracted from values of SNA recorded during the experiment. The SNA signal was then rectified, integrated in 1-s bins, and for the figures was normalized to basal values (% of control). Response values of LSNA, SSNA, MAP, and HR were the difference between the averages of 1 min bins following injection and the 1 min averages of baseline values before the first injection.

All data are presented as means ± SEM. Between group differences were assessed using 2-way repeated measures ANOVA and the *post hoc* Newman–Keuls test (GB-Stat v10, Dynamic Microsystems); *p* < 0.05 was considered statistically significant.

### RNAscope fluorescent FISH

#### Brain sectioning and FISH protocol

Rats were deeply anesthetized with pentobarbital and perfused transcardially with 400- to 500-ml ice-cold isotonic saline, followed by 4% paraformaldehyde (pH 7.4, 100 ml). The brains were removed and postfixed for 6–18 h at 4°C. Brains were sectioned (15–30 μm) and either mounted directly onto Superfrost Plus slides (Fisher Scientific) and stored at −80°C, or placed in cryoprotectant (30% ethylene glycol, 20% glycerol, and 50 mm sodium phosphate buffer, pH 7.4) at −20°C until further processing. Sections stored in cryoprotectant were briefly washed in sterile PBS before mounting on charged slides, and dried overnight. All sections for an experimental “run” were mounted and reacted on the same slide and thus experienced the same experimental conditions and solutions. Sections mounted were selected every 90, 120, 180, or 360 μm (depending on experiment) throughout the ArcN (from −1.92 to −3.60 mm from bregma). After two rinses in sterile water, sections were incubated with protease IV from the RNAscope Multiplex Fluorescent Assay kit [Advanced Cell Diagnostics (ACD); RRID:SCR_012481] for 30 min at 40°C. Sections were rinsed twice in sterile water and incubated in RNAscope catalog oligonucleotide probes (described in Table 1) for 2 h at 40°C. The rest of the FISH was done per manufacturer’s instructions. When more than one probe was incubated simultaneously, different probes were in unique channels and tagged with unique fluorophores.

**Table 1:** List of RNAscope probes

Transcript	Catalog #	Fluorescent tag	Accession #; target region
Npy	450971	Atto 647	NM_012614.2; bp 8–498
Agtr1a	422661	Atto 550	NM_030985.4; bp 1040–2163
Pomc	318511	Atto 647	NM_139326.2; bp 21–921
Kiss1	503421	Alexa Fluor 488	NM_181692.1; bp 14–386
Slc32a1	424541	Atto 647	NM_031782.1; bp 288–1666
Slc17a6	317011	Alexa Fluor 488	NM_053427.1; bp 1109–2024

#### Mapping and imaging

Sections were imaged at 20× on a Zeiss ApoTome2 on AxioImager with a 20 × 0.8 PlanApo objective or 63×, 1.4 (oil) Plan Apo objective. Filter settings for Alexa Fluor 488, Atto 550, and Atto 647 fluorophores were as follows: Alexa Fluor 488, excitation of 500 nm, emission of 535 nm; Atto 550, excitation of 545, emission of 605 nm; Atto 647, excitation of 640 nm, emission of 690 nm. Neurons were plotted with the Neurolucida software (Micro Brightfield; RRID:SCR_001775). Only cell profiles that included a nucleus and more than or equal to three fluorescent grains were counted and/or mapped. Sections were matched as closely as possible to brain levels with reference to bregma using the atlas of Paxinos and Watson (2007) or Paxinos and Watson (2013). Cells were counted and mapped unilaterally. To determine whether AT1aR neurons were close by neurons with other phenotypes, we used the Colocalization function of Neurolucida specifying a distance of either 20 or 25 μm, as indicated in Results.

Two camera systems were used to image the sections. In one, photographs were taken with a Hamamatsu C11440 Orca-Flash 4.0LT digital camera (resolution 2048 × 2048 pixels) and the resulting TIFF files were first exported into Fiji (RRID:SCR_002285) and the unsharp mask filter and/or brightness/contrast were adjusted for clarity and to reflect true rendering as much as possible. Images were not otherwise altered. TIFF images were imported into Canvas v10 (ACD; RRID:SCR_014312) for labeling and final presentation. In the second system, photographs were taken with a Zeiss AxioCam 506 mono camera (2752 × 2208 pixels) using a Apotome.2 grid illumination device (five phase translations per image). Raw image data files were processed with default settings in Zeiss ZEN 2.3 software and the resulting .czi files were adjusted for brightness/contrast for clarity and to reflect true rendering as much as possible. Images were not otherwise altered. Images were analyzed by using the positive and negative controls to set imaging processing parameters and background, respectively.

## Results

### Sympathoexcitatory effects of ArcN AngII in male rats.

There were no differences in the baseline values of MAP and HR between groups (Table 2).

**Table 2:** Baseline values of MAP and HR in male rats

	**ArcN aCSF + AngII** (*n* = 6)	**ArcN candesartan + AngII** (*n* = 5)	**ArcN candesartan + aCSF** (*n* = 4)
MAP (mmHg)	97 ± 4	106 ± 8	105 ± 9
HR (bpm)	327 ± 15	360 ± 19	339 ± 19
			
	**ArcN AngII + PVN BIBO3304** (*n* = 5)	**ArcN AngII + PVN aCSF** (*n* = 5)	**ArcN aCSF + PVN BIBO3304** (*n* = 5)
MAP (mmHg)	106 ± 5	103 ± 4	118 ± 6
HR (bpm)	365 ± 18	371 ± 24	362 ± 27
			
	**ArcN AngII + DMH BIBO3304** (*n* = 4)	**ArcN aCSF + DMH BIBO3304** (*n* = 4)	**AngII + DMH aCSF** (*n* = 4)
MAP (mmHg)	89 ± 6	98 ± 7	92 ± 5
HR (bpm)	355 ± 12	370 ± 23	326 ± 27
			
	**PVN SHU9119 + ArcN AngII** (*n* = 5)	**PVN SHU9119 + ArcN aCSF** (*n* = 5)	
MAP (mmHg)	104 ± 7	99 ± 8	
HR (bpm)	360 ± 27	373 ± 14	

#### ArcN AngII nanoinjections increase SNA by activating AT1aR

**Figure 1.**
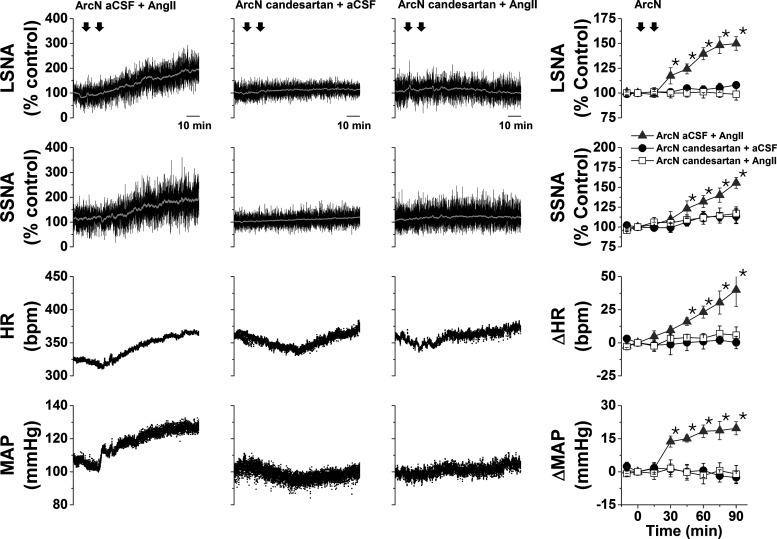
ArcN AngII increases LSNA, SSNA, HR, and MAP via AT1aR in male rats. Representative experiments (left three columns) and grouped data (right column) showing that bilateral nanoinjections of AngII into the ArcN slowly increased LSNA and SSNA (*n* = 6), and this sympathoexcitation was blocked by prior administration of candesartan (*n* = 5). ArcN nanoinjections of candesartan have no significant effects when followed by ArcN injections of aCSF (*n* = 4). The first arrow represents the time of the first ArcN bilateral injection (aCSF or candesartan), and the second arrow represents the time of the second injection (AngII or aCSF). Gray triangles: ArcN aCSF + AngII; black closed circles: ArcN candesartan + aCSF; open squares: ArcN candesartan + AngII; *p < 0.05 compared with baseline (time 0). Error bars represent SEM.

In initial experiments, unilateral ArcN injections of AngII (1 mmol/l) only transiently increased SNA in male rats; therefore, the remaining experiments used bilateral injections. When administered bilaterally, AngII instead produced a slowly developing and sustained increase in LSNA, SSNA, MAP, and HR (Fig. 1). While bilateral ArcN candesartan injections had no effects, candesartan pretreatment completely prevented the responses to injections of AngII into the ArcN 10–15 min later (Fig. 1). Nevertheless, 90 min after ArcN AngII, ArcN candesartan administration failed to significantly reverse AngII-induced sympathoexcitation (*n* = 3; data not shown). Therefore, ArcN AngII activates AT1aR to increase LSNA and SSNA via a poorly reversible mechanism.

#### Role of NPY projections to the PVN and DMH in ArcN AngII-induced sympathoexcitation

**Figure 2.**
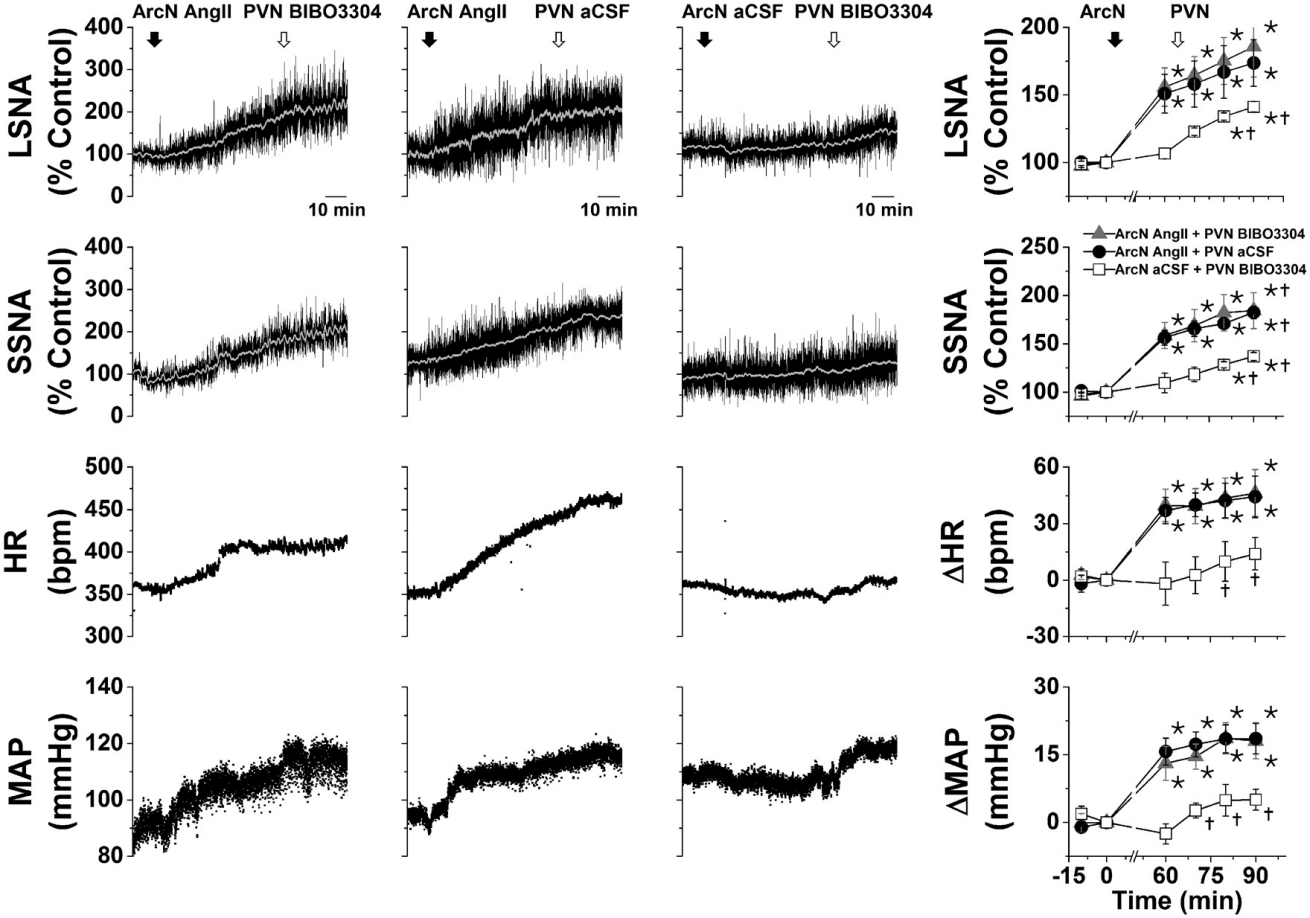
ArcN AngII suppresses tonic PVN NPY sympathoinhibition in male rats. Representative experiments (left three columns) and grouped data (right column) showing that blockade of PVN NPY Y1R increases LSNA and SSNA (*n* = 5; 60 min after ArcN nanoinjections of aCSF); however, ArcN AngII sympathoexcitation was the same whether followed 60 min later by ArcN BIBO3304 (*n* = 5) or ArcN aCSF (*n* = 5). Thus, the sympathoexcitation induced by blockade of PVN NPY Y1R was prevented by prior administration of AngII into the ArcN. The first solid arrow represents the time of ArcN bilateral injections (aCSF or AngII) and the second open arrow represents the time of the second PVN injections (BIBO3304 or aCSF). Gray triangles: ArcN AngII + PVN BIBO3304; black closed circles: ArcN AngII + PVN aCSF; open squares: ArcN aCSF + PVN BIBO3304; *p < 0.05 compared with baseline (time 0); †*p* < 0.05 compared with values just before PVN injections (time 60 min). Error bars represent SEM.

**Figure 3.**
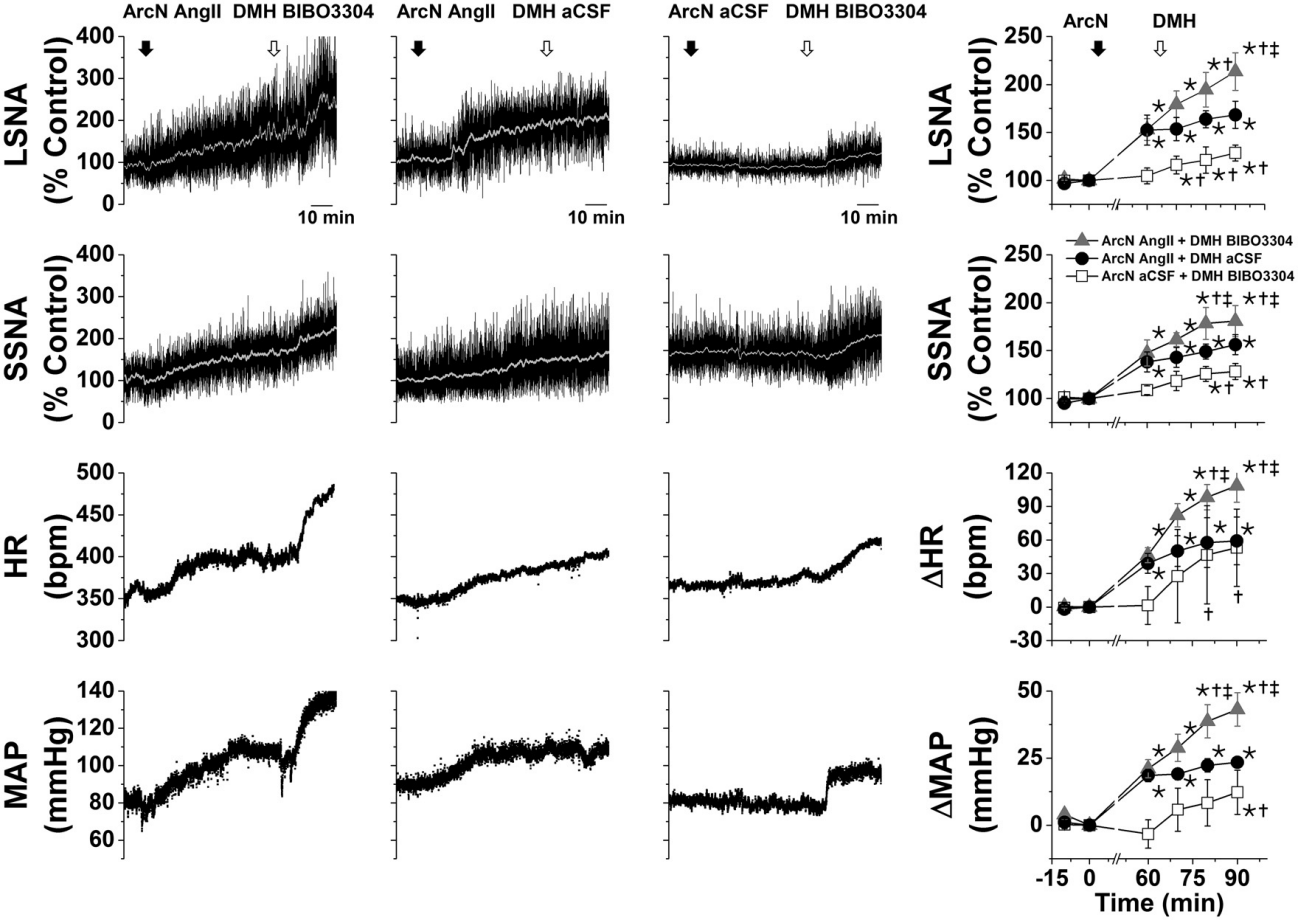
ArcN AngII does not suppress tonic DMH NPY sympathoinhibition in male rats. Representative experiments (left three columns) and grouped data (right column) showing that blockade of DMH NPY Y1R increases LSNA and SSNA (*n* = 4; 60 min after ArcN nanoinjections of aCSF). This DMH BIBO3304 sympathoexcitation was similar to the increases induced by BIBO3304 after ArcN AngII (*n* = 4) and was greater than ArcN AngII followed by DMH aCSF (*n* = 4). Thus, the sympathoexcitation induced by blockade of DMH NPY Y1R was not prevented by prior administration of AngII into the ArcN. The first solid arrow represents the time of ArcN bilateral injections (aCSF or AngII), and the second open arrow represents the time of the second DMH injections (BIBO3304 or aCSF). Gray triangles: ArcN AngII + DMH BIBO3304; black closed circles: ArcN AngII + DMH aCSF; open squares: ArcN aCSF + DMH BIBO3304; *p < 0.05 compared with baseline (time 0); †*p* < 0.05 compared with values just before PVN injections (time 60 min); ‡*p* < 0.05 ArcN aCSF + DMH BIBO3304 versus AngII + DMH aCSF at the same time. Error bars represent SEM.

As expected (Cassaglia et al., 2014, 2016; Shi et al., 2015b), after ArcN aCSF, bilateral PVN injections of the high affinity NPY Y1R antagonist, BIBO3304, produced small but significant increases in LSNA, SSNA, MAP, and HR (Fig. 2), indicating that NPY tonically suppresses SNA via PVN Y1R. However, after ArcN AngII, the increases in these variables were the same following PVN BIBO3304 as following PVN aCSF (Fig. 2), suggesting that ArcN AngII suppresses tonic PVN NPY inhibition. As previously reported in mice (Shi et al., 2017), bilateral injections of BIBO3304 into the DMH also increased LSNA, SSNA, MAP, and HR (Fig. 3). In contrast to the PVN, DMH injections of BIBO3304 60 min after ArcN AngII elicited even further increases in these variables (relative to DMH aCSF). Collectively, these data indicate that ArcN AngII increases LSNA and SSNA, in part, by inhibition of ArcN NPY neurons that tonically suppress the activity of PVN presympathetic neurons via Y1R. On the other hand, these results do not support the hypothesis that ArcN AngII similarly suppresses tonic NPY sympathoinhibition via DMH Y1R, although these data alone do not eliminate a possible action of DMH NPY at other receptor subtypes.

#### Role of PVN MC3/4R

**Figure 4.**
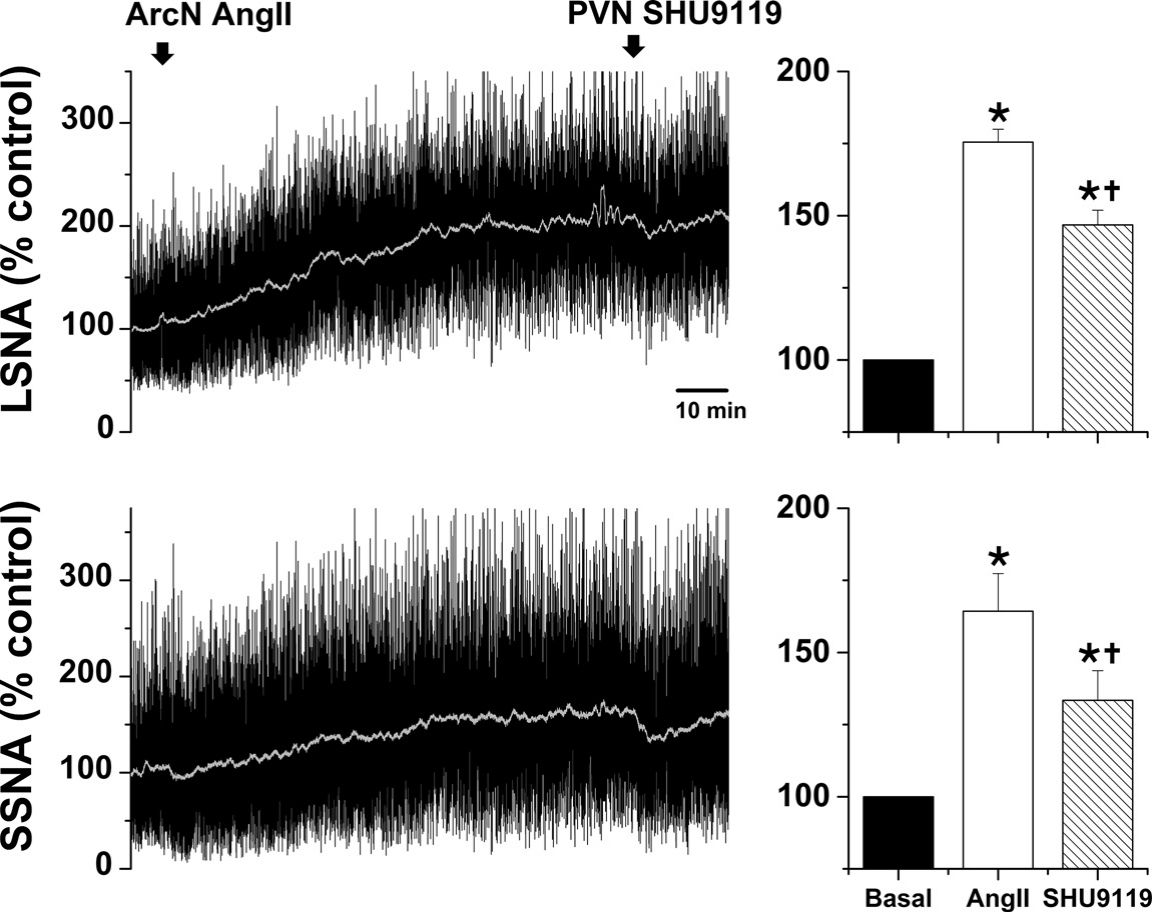
Blockade of PVN MC3/4R with SHU9119 partially reverses the sympathoexcitatory effects of ArcN AngII in male rats. Representative experiments (left) and grouped data (right; *n* = 5) showing that PVN SHU9119 transiently decreases LSNA and SSNA after ArcN AngII. Decreases in MAP and HR were also observed, but these responses did not achieve statistical significance (data not shown); *p < 0.05 compared with baseline (time 0); †*p* < 0.05 compared with values just before PVN injections of SHU9119. Error bars represent SEM.

**Figure 5.**
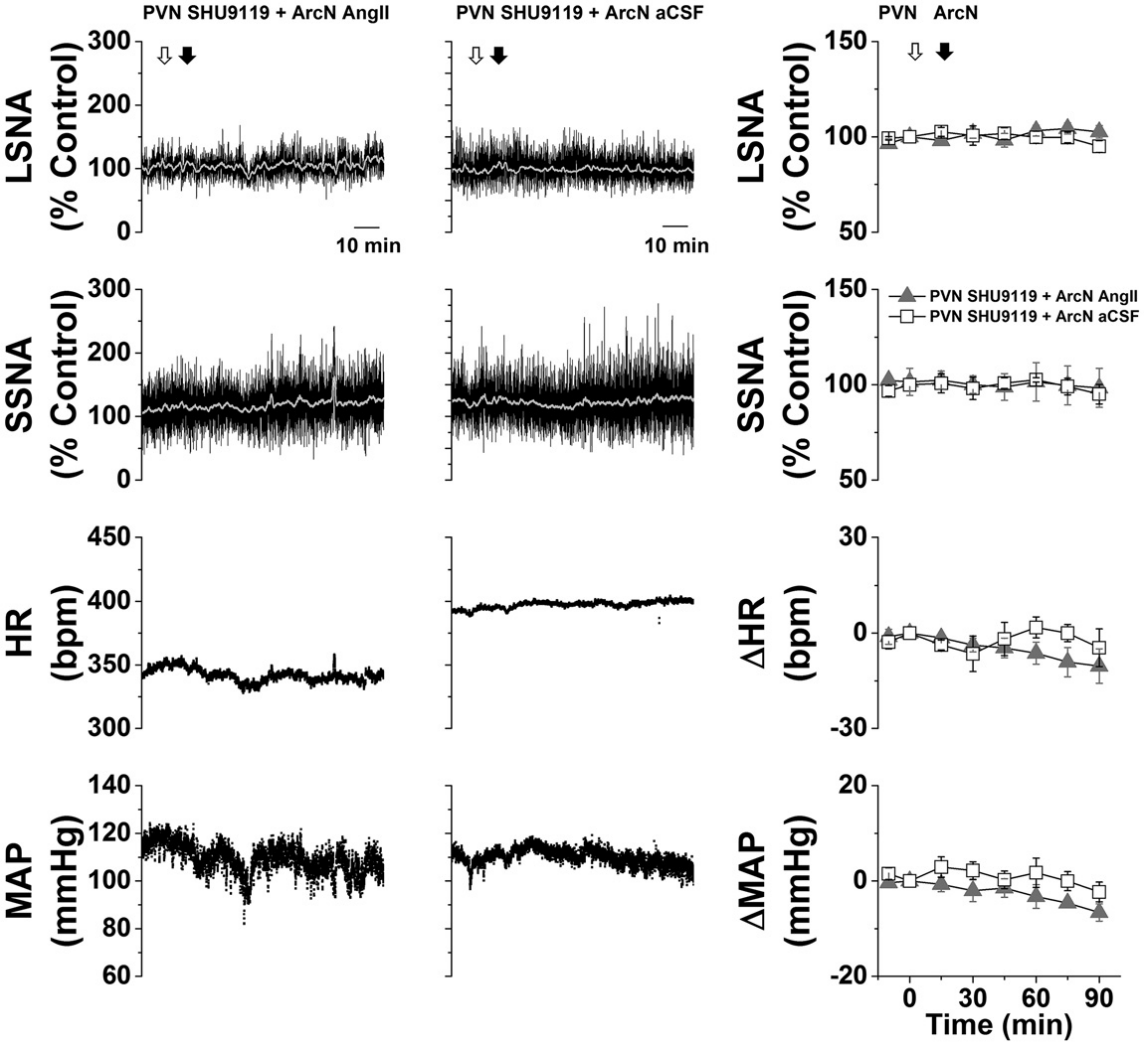
ArcN AngII increases LSNA and SSNA in part by increasing PVN MC4R sympathoexcitation. Representative experiments (left two columns) and grouped data (right column) showing that prior blockade of PVN MC4R with bilateral nanoinjections of SHU9119 completely prevents ArcN AngII sympathoexcitation (*n* = 5), whereas PVN SHU9119 has no effects when followed by ArcN aCSF (*n* = 5). The first open arrow represents the time of PVN bilateral injections of SHU9119 and the second open arrow represents the time of the second ArcN injections (AngII or aCSF). Gray triangles: PVN SHU9119 + ArcN AngII; open squares: PVN SHU9119 + ArcN aCSF. Error bars represent SEM.

In initial experiments, we tested whether PVN administration of the MC3/4R antagonist SHU9119 reversed the increases in LSNA/SSNA after ArcN AngII nanoinjections. PVN SHU9119 did decrease SNA, albeit only partially and transiently (Fig. 4), as previously noted after insulin or in pregnant rats (Ward et al., 2011; Shi et al., 2015a). The failure of PVN SHU9119 to completely reverse the effects of ArcN AngII may be due in part to the induction of MC3/4R (or ArcN AT1aR) signaling that is not rapidly reversed, in parallel to the inability of candesartan to reverse the effects of ArcN AngII. Therefore, we next tested SHU9119 pretreatment. Bilateral injections of SHU9119 into the PVN of rats that subsequently received ArcN aCSF did not alter SNA, MAP, or HR (Fig. 5). However, PVN SHU9119 pretreatment completely prevented the effects of subsequent injection of AngII into the ArcN (Fig. 5). Collectively, these data indicate that ArcN AngII sympathoexcitation relies on POMC projections to the PVN. The ability of PVN SHU9119 to completely block the effects of ArcN AngII, coupled with the failure of ArcN AngII to lessen tonic NPY-Y1R inhibition of the DMH, suggests that ArcN-to-DMH projections of NPY neurons, or parallel excitatory neurons, do not directly participate in the sympathoexcitatory effects of AngII.

#### Histologic verification of injection sites

**Figure 6.**
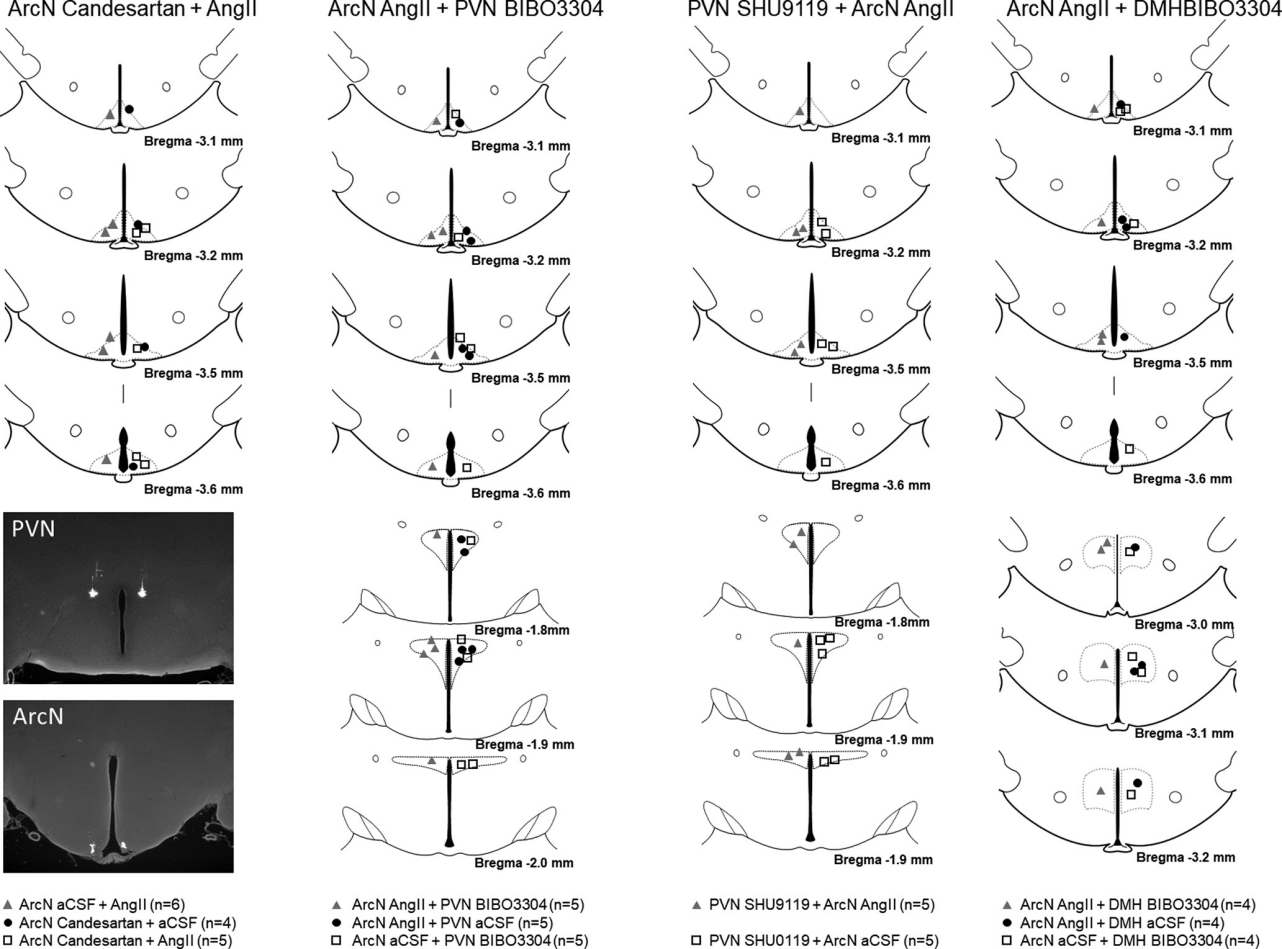
Histologic maps illustrating nanoinjection sites in male rats. Maps adapted from Paxinos and Watson (2007).

Figure 6 illustrates the injection sites for physiological experiments in male rats.

### Sympathoexcitatory effects of ArcN AngII in female rats

#### Basal values (Table 3)

Body weight was similar in rats throughout the estrous cycle. As expected, uterine weight was elevated in rats in proestrus, compared with estrous or diestrous rats. Changes in MAP, HR, LSNA, and SSNA were not detectable during the estrous cycle. However, pregnancy markedly decreased MAP and increased body weight, HR, LSNA, and SSNA.

#### Increases in SNA and MAP in response to ArcN AngII vary during the estrous cycle

**Figure 7.**
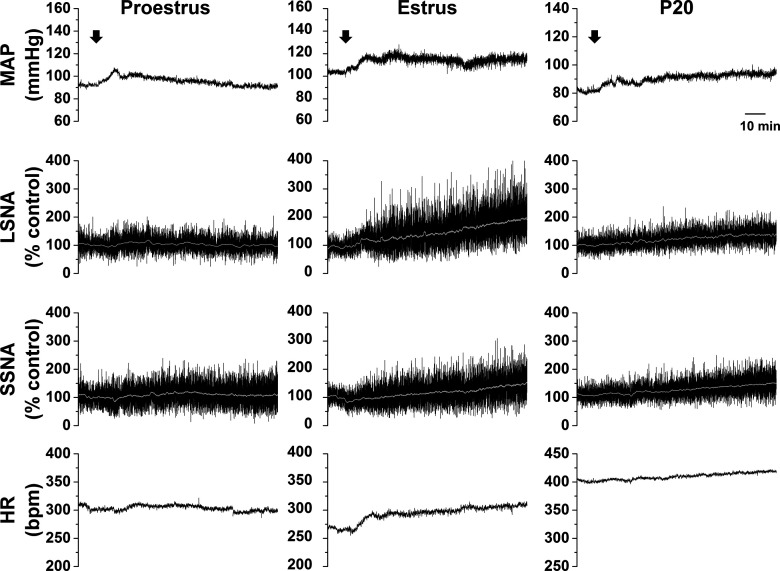
Representative experiments showing that ArcN AngII increases LSNA, SSNA, HR, and MAP during estrus and late pregnancy, but only increases MAP during proestrus. Bilateral nanoinjections of AngII commenced at the arrows.

**Figure 8.**
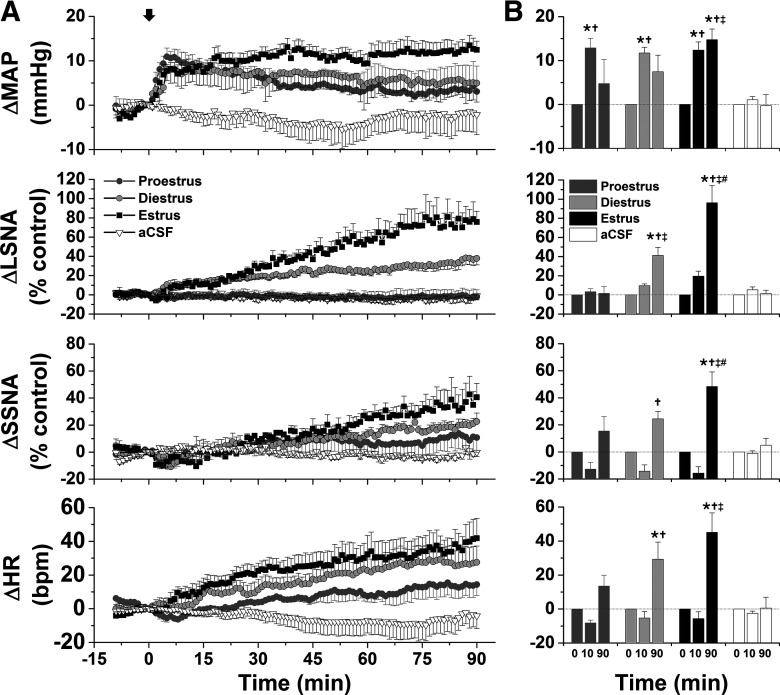
Grouped data showing that ArcN AngII increases LSNA, SSNA, HR, and MAP during estrus and diestrus but only increases MAP during proestrus. **A.** Mean ± SEM of changes in MAP, LSNA, SSNA, and HR in rats during proestrus (dark gray circles, *n* = 5), diestrus (light gray circles, *n* = 4), or estrus (solid black squares, *n* = 6) following bilateral nanoinjections of AngII (beginning at arrow, time 0). Nanoinjections of aCSF are shown by the open triangles (rats in various reproductive stages, *n* = 6). **B.** Statistical comparison of data obtained at baseline (time 0) as well as the maximum changes between 1 and 10 min (time 10 min), and the maximum changes between 81 and 90 min (time 90 min); *compared with time 0; †compared with aCSF at the same time; ‡compared with proestrus at the same time; #compared with diestrus at the same time. Error bars represent SEM.

As shown in representative tracings (Fig. 7) and the grouped data (Fig. 8), bilateral nanoinjections of AngII rapidly (within 10 min) increased MAP in all groups. The pressor response was sustained in estrous rats, but recovered toward baseline in proestrous and diestrous rats. This rapid response is somewhat distinct from that in male rats: while in some males ArcN did elicit an initial pressor response (e.g., representative experiments in Figs. 1-3), overall, the change in MAP within the first 10 min was variable (4.2 ± 2.9 mmHg; *n* = 15; no difference from baseline or females). In females as in males, ArcN AngII also elicited a slowly developing increase in LSNA, SSNA, and HR during estrus and diestrus, but not during proestrus. On the other hand, these variables did not change in cycling rats that received bilateral nanoinjections of aCSF (Fig. 8).

#### Role of AT1aR

Bilateral nanoinjections of candesartan into the ArcN (*n* = 4) had no effects on MAP (−1.6 ± 3.0 mmHg), LSNA (−2.2 ± 2.3% control), SSNA (2.6 ± 2.3% control), or HR (0.7 ± 2.6 bpm). However, the candesartan pretreatment (*n* = 4) prevented ArcN AngII-induced increases (*p* > 0.10–0.90) in MAP (in mmHg: 1.1 ± 2.9, 10 min; −1.6 ± 8.6, 90 min), LSNA (in % control: 3.5 ± 6.2, 10 min; 5.8 ± 10.3, 90 min), SSNA (in % control: 7.6 ± 5.7, 10 min; 14.5 ± 8.7, 90 min), and HR (in bpm: −0.9 ± 5.7, 10 min; 3.6 ± 14.8, 90 min). Therefore, the cardiovascular and sympathoexcitatory effects of ArcN AngII are mediated by AT1aR in females as in males.

#### ArcN AngII engages NPY and POMC projections to the PVN

**Figure 9.**
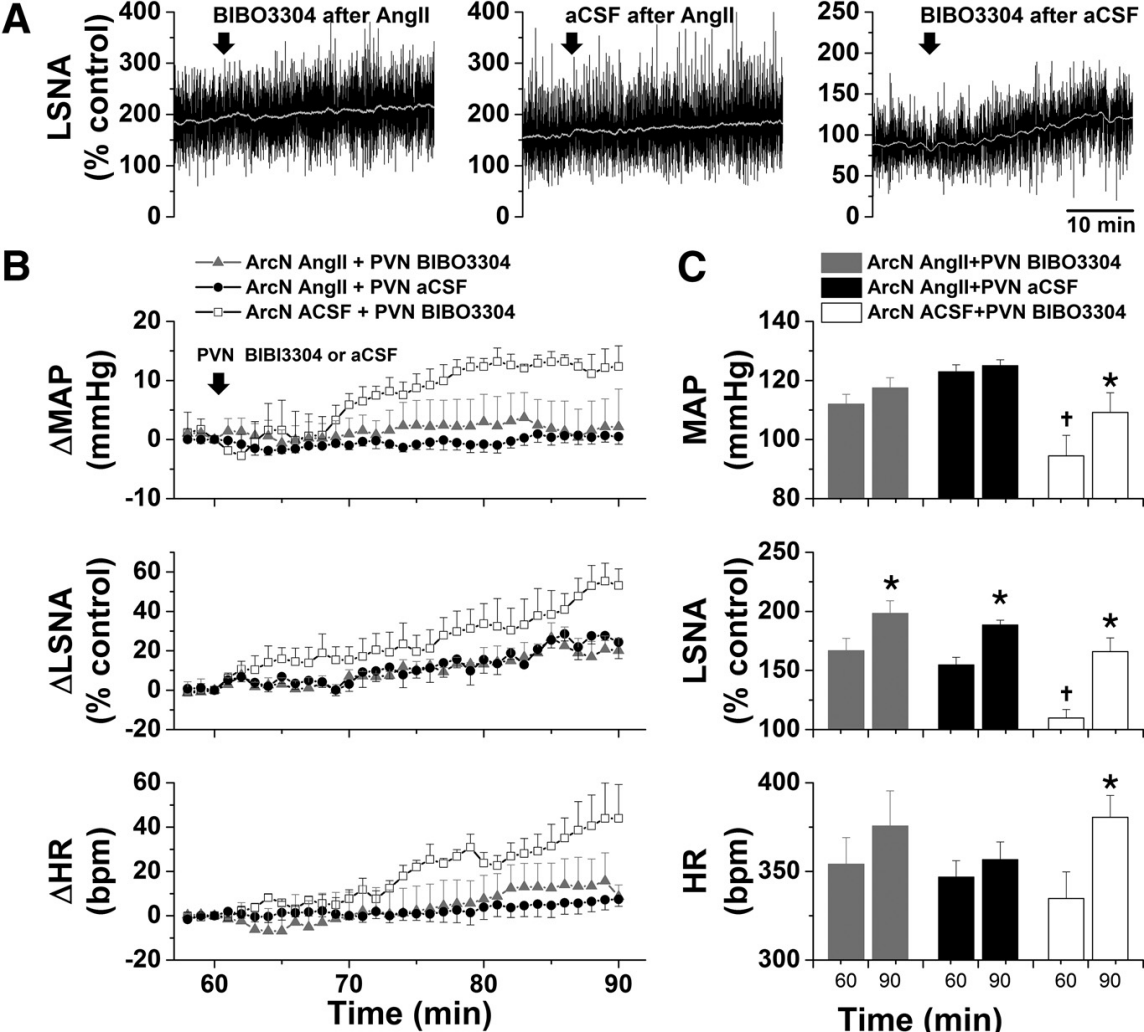
ArcN AngII suppresses tonic PVN NPY sympathoinhibition in female rats. ***A***, Representative experiments showing that blockade of PVN NPY Y1R increases LSNA (right, 60 min after ArcN nanoinjections of aCSF). However, 60 min after ArcN AngII, the increases in LSNA were similar following nanoinjections of BIBO3304 (middle) or ArcN aCSF (left). ***B***, Grouped time course data. The solid arrows represent the time of PVN bilateral injections of BIBO3304 or aCSF, 60 min after ArcN nanoninjections of AngII or aCSF. Open circles: ArcN AngII + PVN aCSF (*n* = 4); gray closed circles: ArcN AngII + PVN BIBO3304 (*n* = 4); closed black squares: ArcN aCSF + PVN BIBO3304 (*n* = 4). Thus, the sympathoexcitation induced by blockade of PVN NPY Y1R was prevented by prior administration of AngII into the ArcN. ***C***, Statistical comparison of data 60 min after ArcN injections (just before PVN injections) and 90 min after ArcN injections (30 min after PVN injections); *p < 0.05 compared with preinjection (60 min after injection of AngII or aCSF at time 0); †*p* < 0.05, at 60 min, values for ArcN aCSF + PVN BIBO3304 are less than for ArcN AngII + PVN aCSF and ArcN AngII + PVN BIBO3304. Error bars represent SEM.

As shown in a representative experiment and grouped data in Figure 9, following ArcN aCSF, blockade of PVN NPY Y1R with bilateral nanoinjections of BIBO3304 increased MAP, LSNA, and HR, indicating that NPY projections to the PVN tonically inhibit these variables. However, 60 min following ArcN AngII, PVN BIBO3304 did not alter MAP and HR; LSNA continued to increase (by 30 ± 6% control), but the increase was the same as after PVN injections of aCSF (by 34 ± 3% control) and significantly smaller (*p* < 0.05) than the increases in LSNA (56 ± 9% control) induced by PVN BIBO3304 after ArcN injections of aCSF. These data suggest that ArcN AngII suppresses tonic NPY inhibition of PVN presympathetic neurons in female as in male rats.

**Figure 10.**
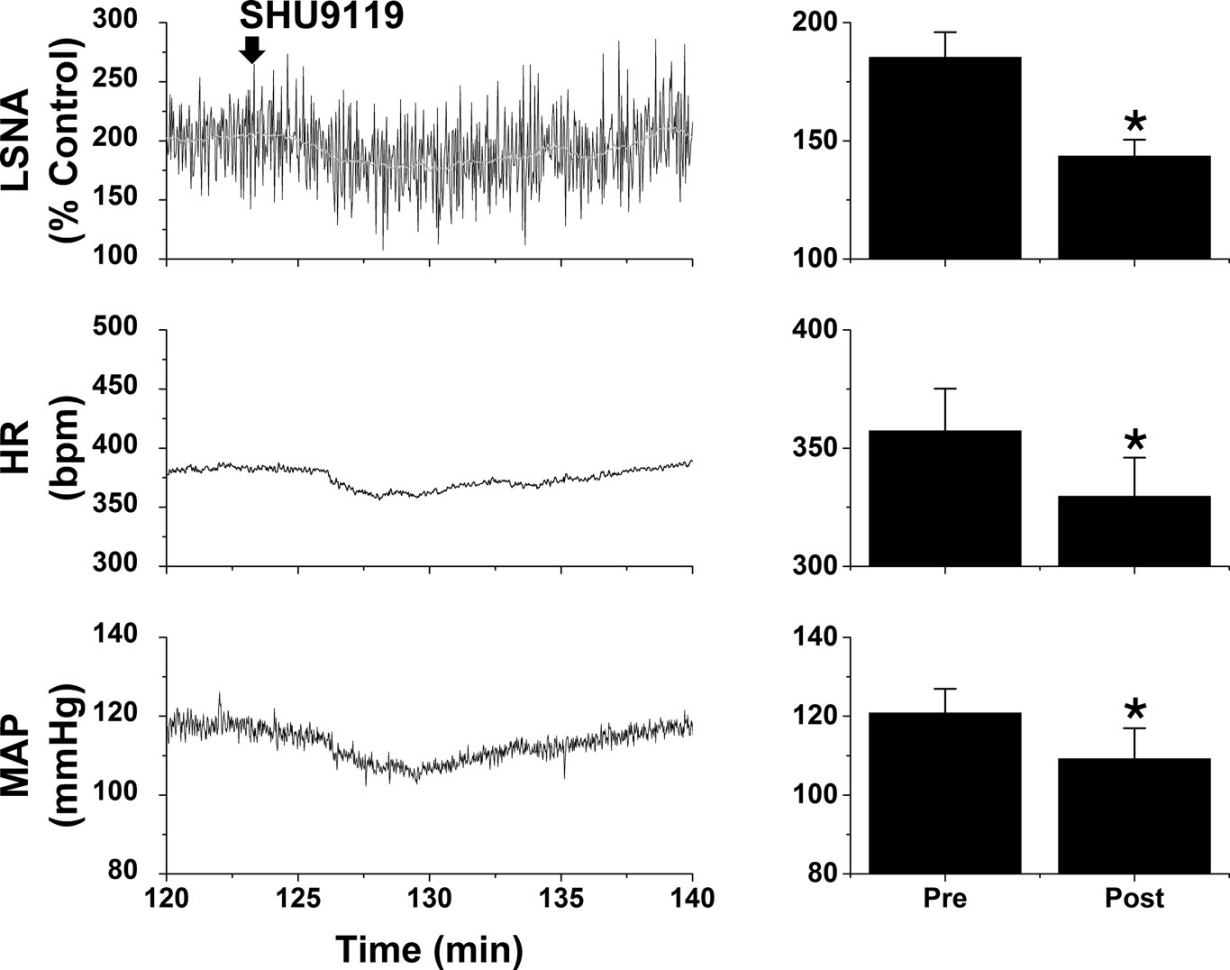
Blockade of PVN MC3/4R with SHU9119 partially reverses the sympathoexcitatory effects of ArcN AngII in female rats. Representative experiments (right) and grouped data (*n* = 4) showing that PVN SHU9119 transiently decreases LSNA, HR, and MAP after ArcN AngII; *p < 0.05 compared with baseline (time 0). Error bars represent SEM.

In separate experiments in rats in estrus (Fig. 10), blockade of PVN MC3/4R with SHU9119, 2 h after ArcN nanoinjections of AngII, decreased LSNA, HR, and MAP. Because PVN SHU9119 does not alter these variables in otherwise untreated virgin female (Shi et al., 2015a, b) and male rats (Fig. 5), we conclude that, as in males, ArcN AngII increases LSNA at least in part by activating ArcN POMC neurons that release α-MSH in the PVN.

#### ArcN AngII increases MAP and SNA in late pregnant rats

**Figure 11.**
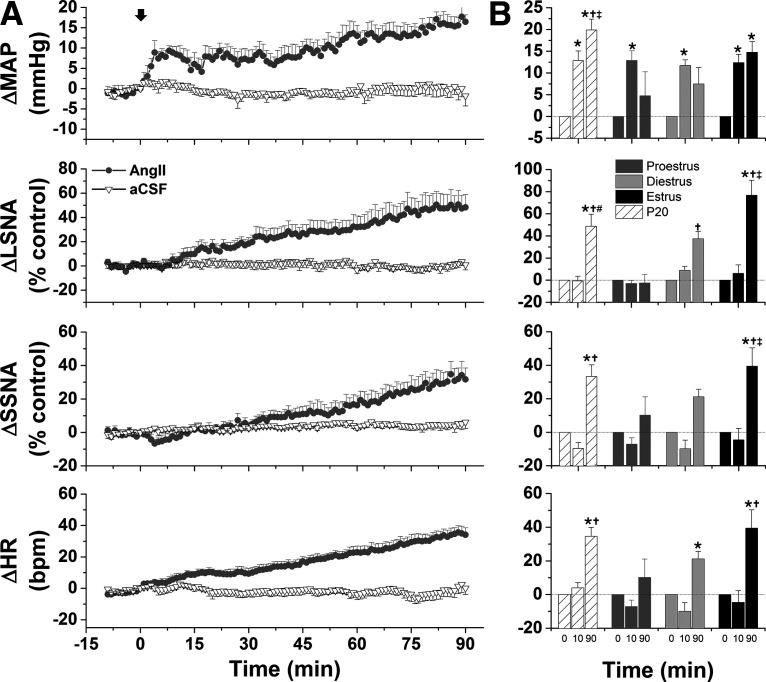
ArcN AngII increases LSNA, SSNA, MAP, and HR in late pregnant rats. ***A***, Grouped time course data showing that bilateral nanoinjections of AngII, but not aCSF (at arrow), increase MAP, LSNA, SSNA, and HR in late pregnant rats (P20; *n* = 6). ***B***, Statistical comparison of data from late pregnant rats (*n* = 6) to cycling rats (data and *n* same as Fig. 8) obtained at baseline (time 0) as well as the maximum changes between 5 and 10 min (time 10 min), and the maximum changes between 88 and 90 min (time 90 min) after injecting AngII or aCSF; *compared with time 0; †compared with proestrus at the same time; ‡compared with diestrus at the same time; #compared with estrus at the same time. Error bars represent SEM.

As in cycling rats, ArcN AngII immediately increased MAP in late pregnant rats (Fig. 11). Like rats in estrus, but not in diestrus or proestrus, the pressor response was sustained for 90 min (Fig. 11). During pregnancy, ArcN AngII also slowly increased LSNA, SSNA, and HR (Fig. 11). Figure 11*B* compares these responses to those from cycling female rats. The initial pressor response was similar between groups, but at the end of the 90 min observation period, the increase in MAP was greater in pregnant rats compared with diestrous or proestrous rats. The increase in LSNA (% of control) was smaller in pregnant compared with estrous rats; however, the absolute LSNA baseline was higher during pregnancy (Table 3), and LSNA increased to similar absolute levels in pregnant and estrous rats (estrus, 2.6 ± 0.6 μV; P20, 4.7 ± 0.3 μV; NS (not significant)). Therefore, the % change may have been smaller, because LSNA in pregnant rats started from a higher baseline and the AngII-induced increases reached similar maximal absolute levels in both groups. On the other hand, the increase in LSNA induced by ArcN AngII in pregnant rats, was greater than proestrous rats, but similar to diestrous rats (Fig. 11*B*). ArcN AngII increased SSNA (% control) and HR similarly during pregnancy and estrus, but more than during proestrus and diestrus (Fig. 11*B*).

**Table 3:** Effect of pregnancy and the reproductive cycle on MAP, HR, SNA, and uterine weight

	Proestrus	Diestrus	Estrus	P20
Number of rats	7	5	30	24
BW (g)	280 ± 6	273 ± 14	271 ± 3	408 ± 6*
MAP (mmHg)	105 ± 6	98 ± 6	107 ± 2	81 ± 2*
HR (bpm)	326 ± 9	333 ± 15	317 ± 5	379 ± 6*
LSNA (μV)	1.2 ± 0.4	0.8 ± 0.3	1.2 ± 0.1	3.7 ± 0.4*
SSNA (μV)	1.3 ± 0.4	1.9 ± 0.6	1.1 ± 0.2	3.6 ± 0.3*
Uteri weight (g)	0.84 ± 0.04**^†^**	0.45 ± 0.06	0.59 ± 0.02	NA
Litter size				10–17

*p < 0.05, difference between P20 and the other groups; †*p* < 0.05, proestrus different from diestrus and estrus.

#### Vasopressin contributes to the pressor response to ArcN AngII in estrous and pregnant rats

**Figure 12.**
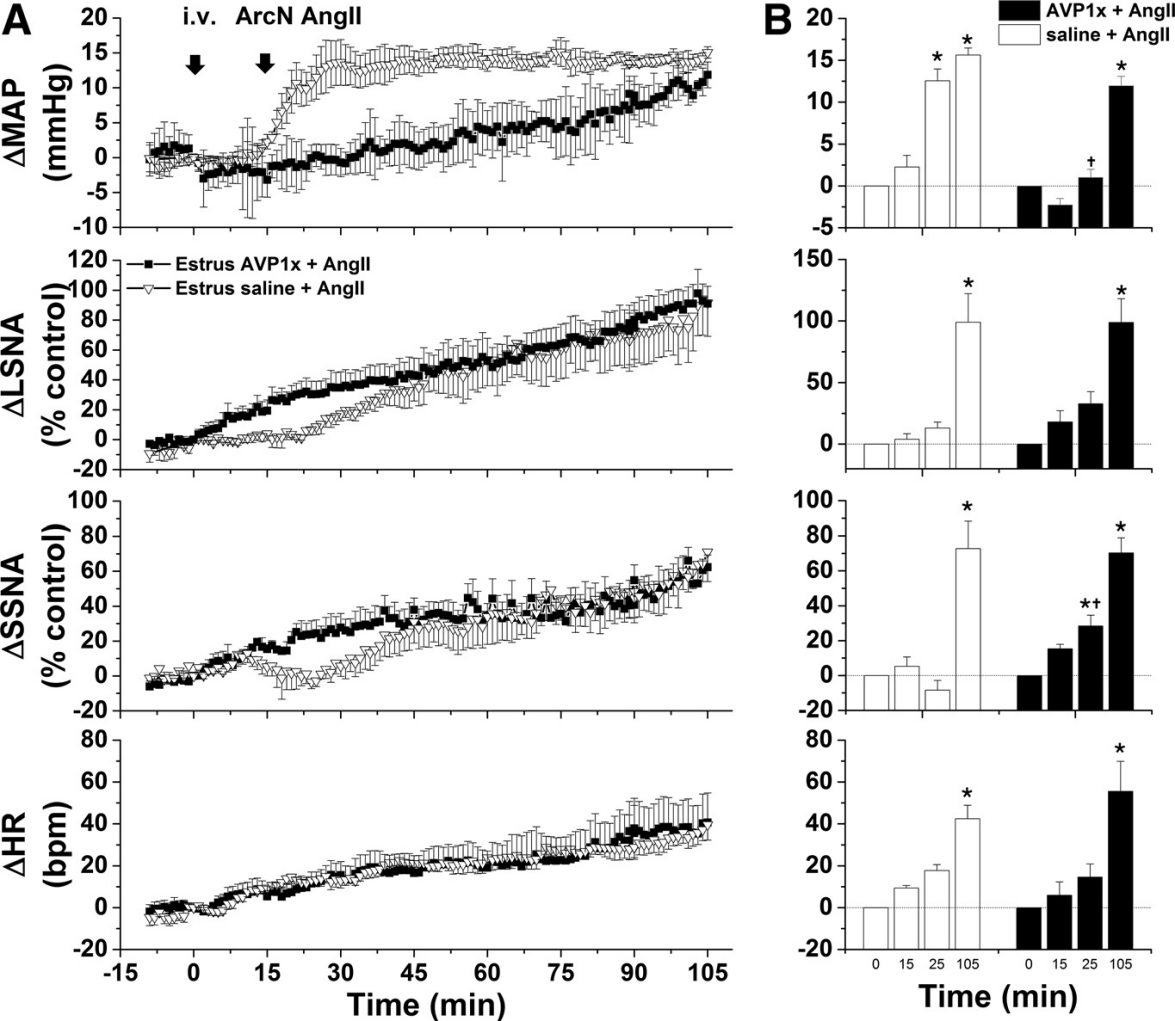
The initial pressor response to ArcN AngII in estrous rats is mediated by increased vasopressin secretion. ***A***, Grouped time course data from rats in estrus showing that the initial, rapid increase in MAP triggered by ArcN AngII nanoinjections 15 after intraperitoneal injections of saline (open triangles; *n* = 5) are abolished 15 min after intraperitoneal injections of the AVP type 1 receptor antagonist (AVP1x; closed squares; *n* = 4). ***B***, Statistical comparison of data obtained at baseline (time 0), 15 min after intraperitoneal saline or AVP1x, as well as the maximum changes between 15 and 25 min (time 25 min) and the maximum changes between 95 and 105 min (time 105 min) after injecting AngII into the ArcN; *compared with time 0; †between groups at the same time. Error bars represent SEM.

ArcN AngII produced a rapid increase in MAP in all groups of females, before significant increases in SNA or HR, suggesting a hormonal mediator may be involved. Recently, ArcN neurokinin B neurons were shown to project to and regulate vasopressin neurons in the supraoptic nucleus (Pineda et al., 2016). Therefore, we tested the role of vasopressin in the initial pressor response. In estrous rats, the AVP V1aR antagonist given iv had no effects on baseline MAP, LSNA, SSNA, and HR, and these variables remained stable in rats given ArcN aCSF (data not shown). However, AVPV1x pretreatment abolished the early, but not the late increase in MAP induced by ArcN AngII (Fig. 12). After intravenous V1ax, the initial increase in SSNA was greater; however, neither the early nor late changes in LSNA or HR were significantly altered by systemic blockade of AVP V1aR (Fig. 12).

**Figure 13.**
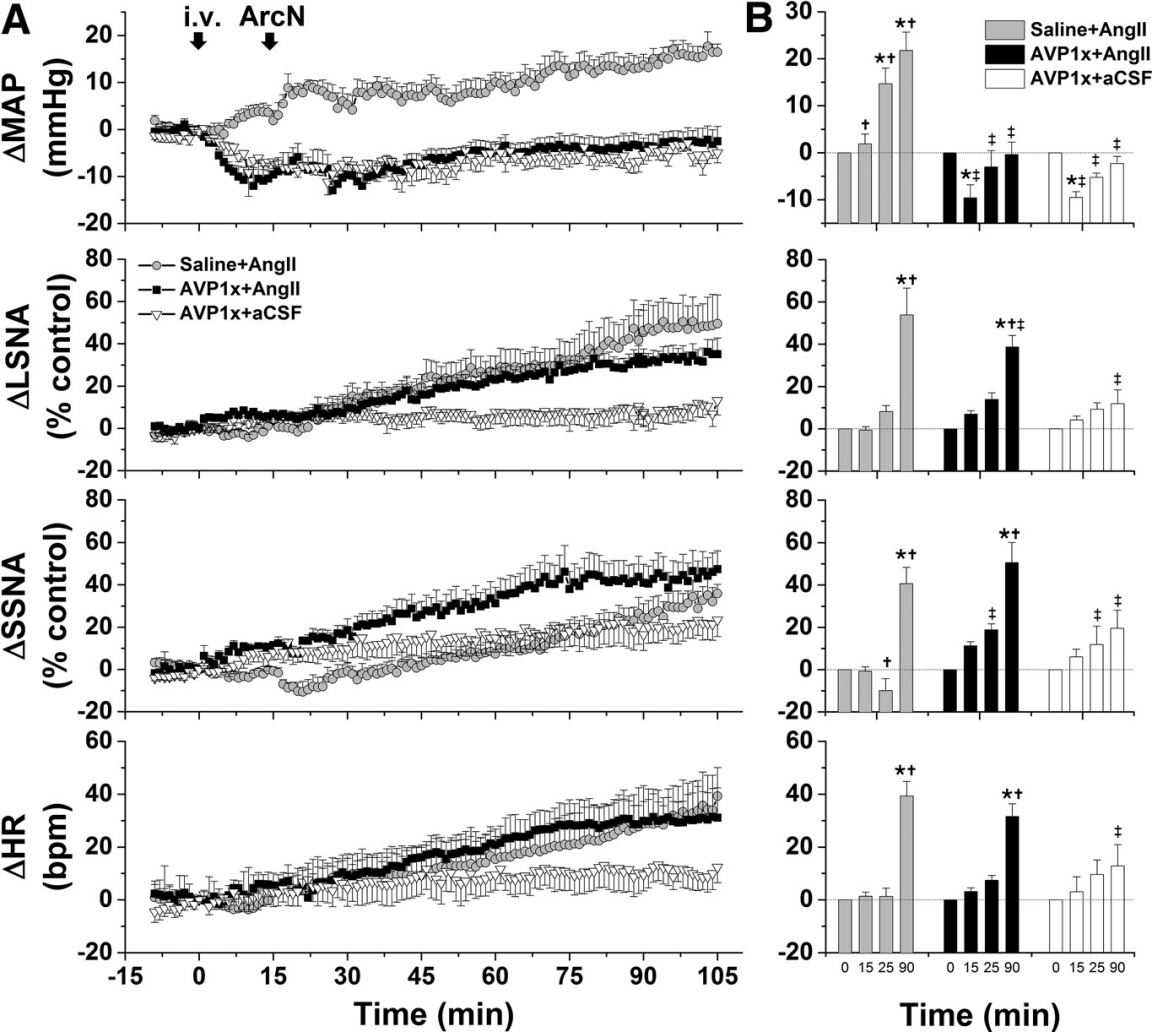
The initial pressor response to ArcN AngII in pregnant rats is mediated by increased vasopressin secretion. ***A***, Grouped time course data from pregnant rats showing that the initial, rapid increase in MAP triggered by ArcN AngII nanoinjections 15 min after intraperitoneal saline (gray circles; *n* = 6) are abolished 15 min after intraperitoneal injections of the AVP type 1 receptor antagonist (AVP1x; closed black squares; *n* = 6). Another group of control pregnant rats received ArcN aCSF 15 min after intraperitoneal AVP1x (open triangles; *n* = 5). ***B***, Statistical comparison of data obtained at baseline (time 0), 15 min after intraperitoneal saline or AVP1x, as well as the maximum changes between 15 and 25 min (time 25 min) and the maximum changes between 95 and 105 min (time 105 min) after injecting AngII into the ArcN; *compared with time 0; †between groups at the same time. Error bars represent SEM.

During pregnancy, intravenous V1ax significantly decreased MAP, which slowly returned to baseline over the 90 min protocol (Fig. 13). This pretreatment totally abolished the ArcN AngII pressor response; after intravenous AVP1x, the change in MAP in rats that received ArcN nanoinjections of AngII was the same as in rats that received ArcN aCSF. An initial AngII-induced decrease in SSNA was transformed into a significant increase following V1ax; however, the increase in HR was unaltered. The increase in LSNA following ArcN AngII was largely unchanged by blocking systemic AVP V1aR, although ultimately a lower level was achieved in this group. These data indicate that during pregnancy, AngII-induced vasopressin release completely mediates the pressor response induced by ArcN AngII, without a significant contribution from the parallel increases in SNA, likely because of reduced vascular responsiveness to norepinephrine.

#### Histologic verification of injection sites

**Figure 14.**
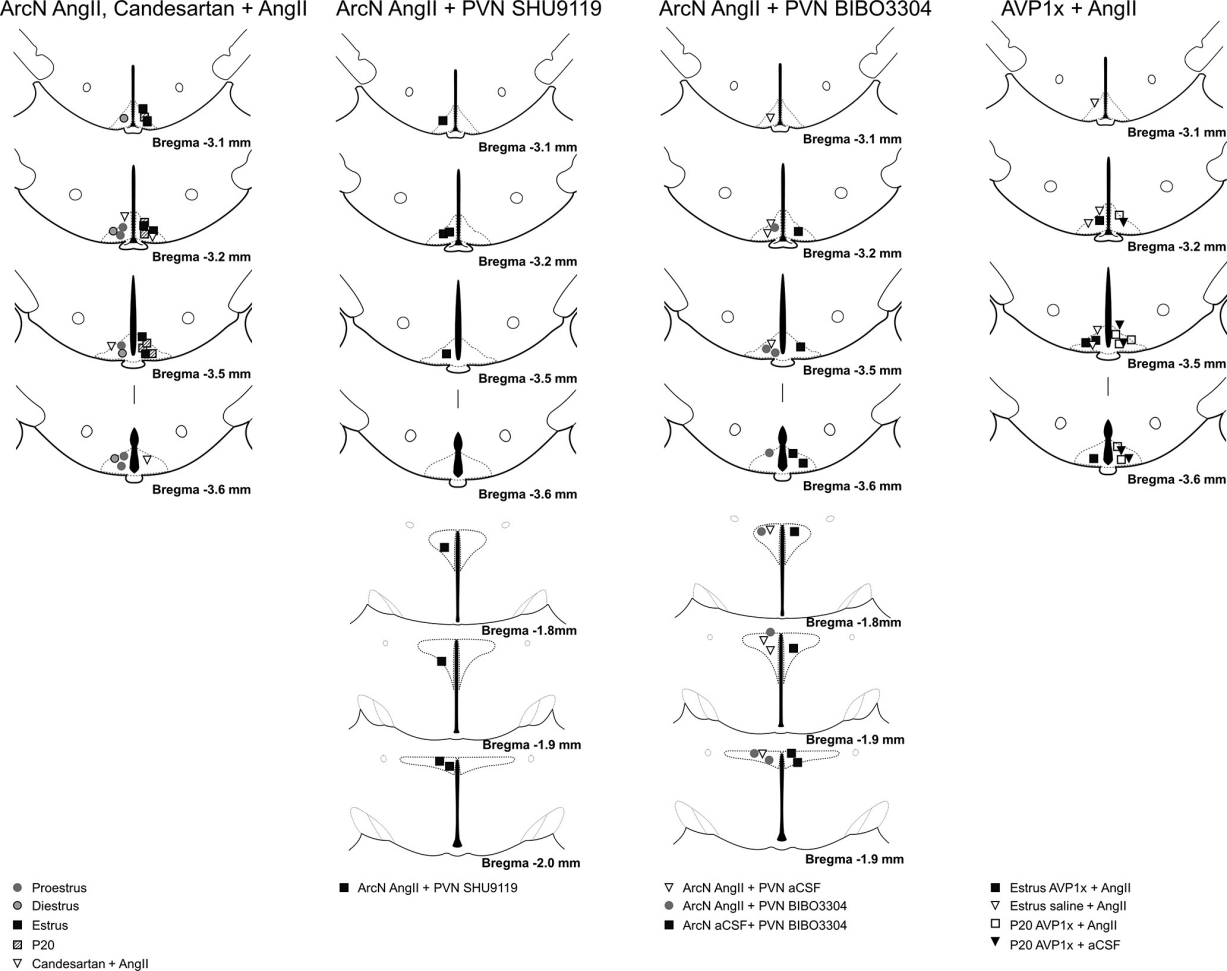
Histologic maps illustrating nanoinjection sites in female rats. Maps adapted from Paxinos and Watson (2007).

Figure 14 summarizes the ArcN and PVN nanoinjections sites for the experiments in female rats.

### ArcN AT1aR expression profiles in male and female rats

Our data show that stimulation of ArcN AngII AT1aR increases SSNA, LSNA, MAP, and HR in male rats and in female rats that are pregnant or in estrus/diestrus; in proestrous rats, AngII only increases MAP. The autonomic responses depend on POMC/α-MSH activation and simultaneous suppression of tonic NPY-mediated inhibition, of preautonomic neurons in the PVN. In females, we further show that the initial pressor response is mediated by increased vasopressin secretion. To begin to understand the cellular mechanisms, we next performed a comprehensive survey of ArcN AT1aR expression in male and female rats.

**Figure 15.**
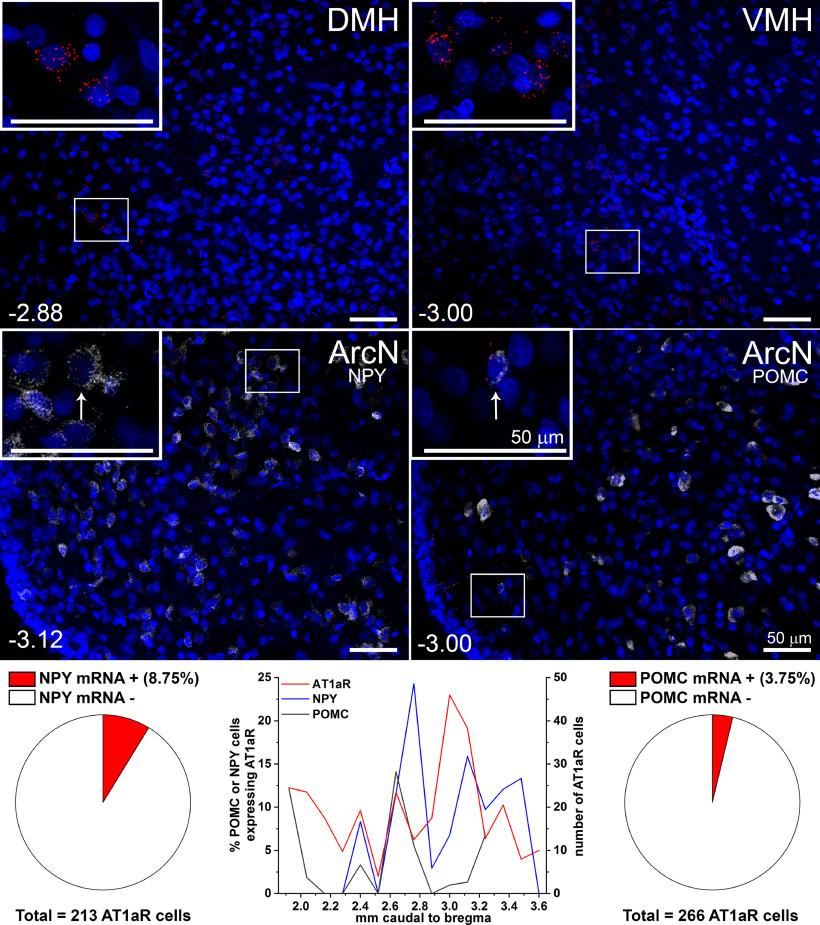
AT1aR are expressed in the ArcN of male rats, albeit at low levels. In male rats, AT1aR are expressed throughout the ArcN (middle and bottom panels) at low levels compared with the DMH and VMH (top panel). A small fraction (8.75%) of AT1aR-positive cells also express NPY and an even smaller fraction (3.75%) are POMC neurons. Bregma levels in lower left corners. Scale bars: 50 μm.

Previous studies in male rats that employed autoradiography or ISH were unable to detect AT1aR expression in the ArcN (Jöhren et al., 1997; Lenkei et al., 1997). Therefore, we used RNAscope, which amplifies expression, to examine AT1aR throughout the ArcN in males. As expected, the signal was weak, compared with neighboring hypothalamic nuclei, like the DMH or VMH (Fig. 15), but clearly evident. AT1aR-positive cells were observed in all levels of the ArcN, but were particularly prominent in the mid-to-caudal segments (Fig. 15). Similarly to mice (Claflin et al., 2017), AT1aRs were expressed in NPY neurons, albeit at a lower level (9%; Fig. 15). AT1aR expression was also detectable in POMC neurons, but rarely (Fig. 15). Thus, in male rats, most AT1aR-expressing cells were neither NPY nor POMC neurons.

**Figure 16.**
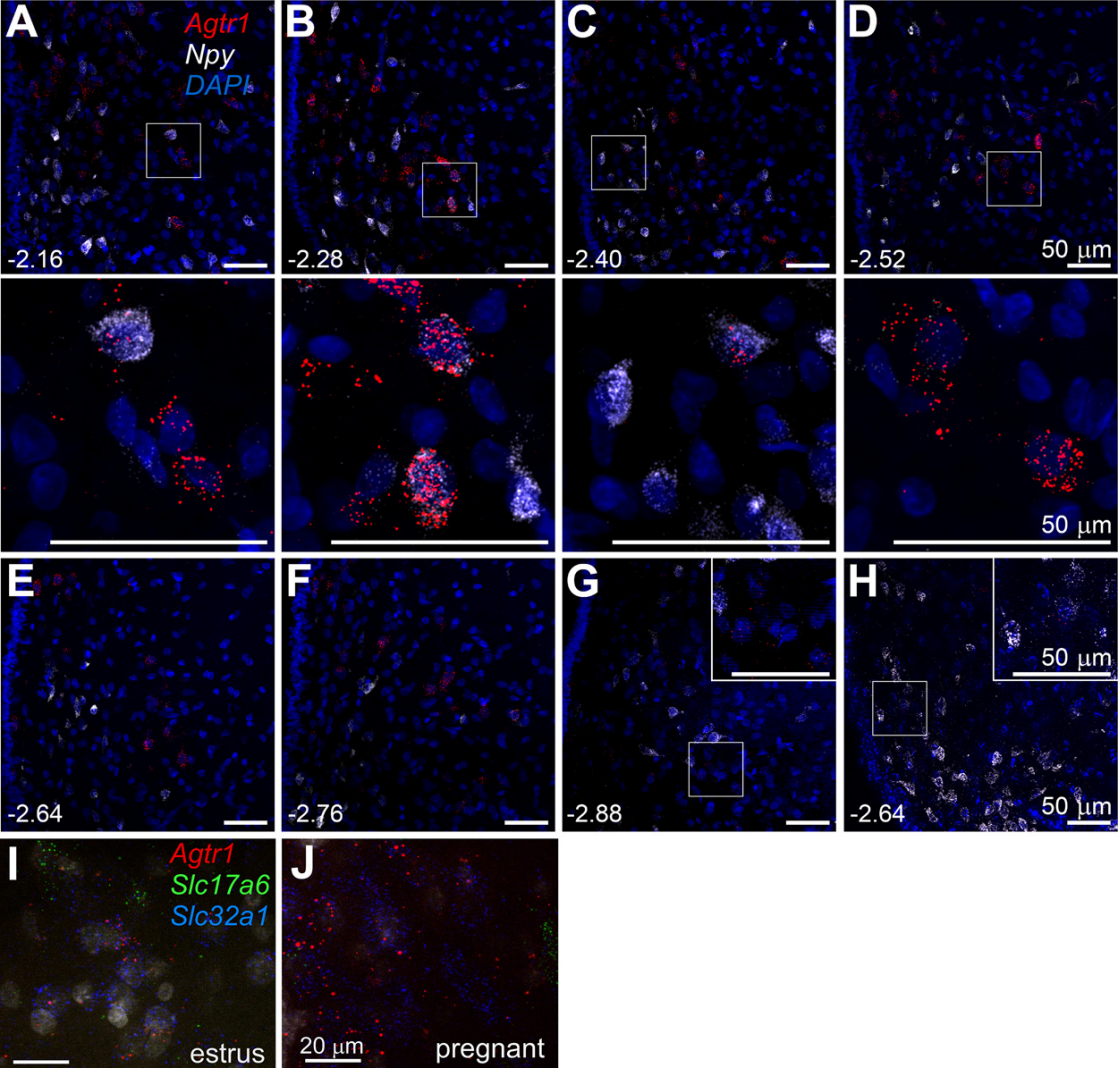
AT1aR are highly expressed in the ArcN during pregnancy. ***A–F***, Sections from a representative pregnant rat various levels throughout the ArcN (mm behind bregma shown in lower left corner) showing high expression levels of the AT1aR (red puncta) compared with rats in diestrus (***G***; low signal, similar to males), proestrus (***H***; almost undetectable as in Jöhren et al. (1997), or estrus (***I***; higher than diestrus/proestrus, but not as high as pregnancy). The images in the second panel are enlarged from the boxed areas directly above (***A–D***). Insets in G and H are enlargements from the boxed areas in each image. As in males, the limited colocalization of AT1aR (red) with NPY (white; ***A–H***) appears in the mid-ArcN levels. AT1aR mRNA (Agtr1, red) colocalizes more often with VGAT mRNA *(Slc32a1*, blue) than with Vglut2 mRNA (*Slc17a6*, green) in both estrus (***I***) and pregnant rats (***J***). Thus, most AT1aR in the rat ArcN is expressed in VGAT, non-NPY non-POMC, neurons. Scale bars: 50 μm (***A–H***) and 20 μm (***I***, ***J***).

In females, we confirm (Jöhren et al., 1997) that ArcN AT1aR expression varies throughout the reproductive cycle, with the highest levels observed in estrus compared with diestrus; proestrus AT1aR expression was nearly undetectable, even using RNAscope (Fig. 16). As in males, AT1aR expression was observed throughout the rostral to caudal ArcN (Fig. 16). A major novel finding, however, was that ArcN AT1aR expression increased dramatically during pregnancy [estrus (*n* = 3) with 73 ± 11 AT1aR-positive cells counted in five sections per animal; pregnancy (*n* = 3) with 121 ± 7 AT1aR-positive cells counted in five equivalent sections; *p* < 0.05; Fig. 16], with the greatest increases observed in the more caudal levels (data not shown). As a result, much of the rest of our analysis was conducted in pregnant rats.

**Figure 17.**
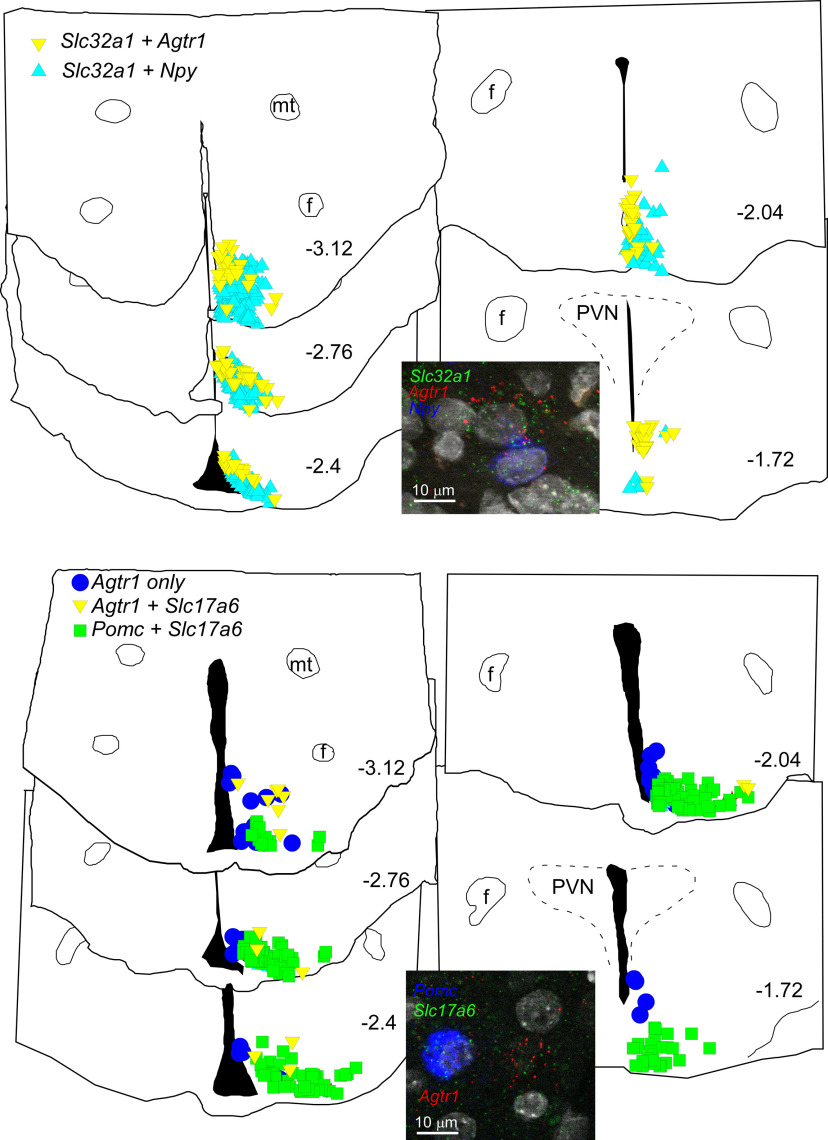
The distribution of AT1aR (*Agtr1*) + VGAT (*Slc32a1*) neurons overlaps with NPY neurons, but AT1aR neurons have little overlap with the distribution of POMC neurons in the ArcN; in females, Agtr1 is not expressed in POMC (*Pomc*) neurons in the ArcN. Top, Computer stage-assisted drawings of hypothalamic coronal sections showing the distribution of *Agtr1 + Slc32a1* (yellow inverted triangles) and *Agtr1 + Npy* (light blue triangles) in the ArcN (upper drawings). Inset, Photomicrograph of the ArcN showing RNAscope assay in upper panel for *Agtr1* (red dots), *Slc32a1* (green dots) and *Npy* (blue dots). Bottom, Computer stage-assisted drawings of hypothalamic coronal sections showing the distribution of *Agtr1* only (blue circles), *Agtr1 + Slc17a6* (yellow inverted triangles) and *Pomc + Slc17a6* (green squares) in the ArcN (lower drawings). Inset, Photomicrograph of ArcN showing RNAscope assay in lower panel for *Agtr1* (red dots), *Slc17a6* (green dots) and *Pomc* (blue dots). Scale bar: 10 μm (both photomicrographs). Approximate millimeters behind bregma (after Paxinos and Watson, 2013) indicated by numbers in lower right of each section. PVN, paraventricular nucleus and as in Figure 16.

We first examined co-localization of AT1aR with NPY or POMC in pregnant rats. Similarly to males, a small percentage of AT1aR-positive cells also express NPY (11.7 ± 5.7%, *n* = 3 pregnant rats, 9 sections per rat; Fig. 16). However, AT1aR expression in POMC neurons was undetectable (*n* = 3, 690 total POMC neurons, counted in 3 rats, 9 sections per rat; Fig. 17, bottom). Thus, as in male rats, most AT1aR-expressing cells were neither NPY nor POMC neurons, which suggests that ArcN AngII inhibits NPY neurons and activates POMC neurons indirectly.

Therefore, we next determined whether AT1aR-expressing neurons were glutamatergic or GABAergic, using the markers Slc17a6 (VGlut-2) and Slc32a1 (VGat). The vast majority of AT1aR neurons were GABAergic, in both estrus (see Visual Abstract) and pregnant (Fig. 17) rats. For example, in pregnant rats, 79 ± 6% AT1aR-positive neurons also expressed VGat (*n* = 7), whereas only 15 ± 4% expressed VGlut-2 (*n* = 8). In estrous rats, 86% of AT1aR cells also expressed VGat and 11% were VGlut-2 (*n* = 2). To test whether activation of ArcN AT1aR could inhibit NPY via a GABAergic interneuron, we determined whether cells that express the AT1aR are nearby inhibitory NPY+VGat neurons. Indeed, cells that expressed the AT1aR spatially overlapped with these NPY+VGat neurons (Fig. 17, top). This was especially apparent in the more caudal portions of the ArcN where, at 3.12 mm caudal to bregma, 37 ± 10% of AT1aR-expressing VGat neurons counted in 3 rats were within 20 μm of NPY+VGat-expressing cells. For the levels corresponding to 2.76, 2.4, 2.04, and 1.76 mm caudal to bregma, these percentages were 23 ± 4%, 27 ± 17%, 15 ± 8% and 0, respectively. Therefore, these anatomic data predict that ArcN AngII could inhibit NPY neurons via an AT1aR-expressing and GABA-releasing interneuron.

To test whether AT1aR-expressing neurons could activate POMC neurons via glutamate release, we also determined how commonly AT1aR-VGlut-2 neurons were nearby POMC neurons (within 25 μm). However, in contrast to the frequent association of AT1aR cells with NPY neurons, POMC neurons were largely separated from AT1aR-VGlut-2 neurons (<1% within 25 μm, *n* = 3; Fig. 17, bottom). During this analysis, we also found that almost all POMC neurons expressed VGlut-2 (94 ± 5%, *n* = 3; Fig. 17, bottom), which is higher than previously reported (Mercer et al., 2013; Wittmann et al., 2013; Stincic et al., 2018). This higher detected expression level may be explained by the sex (pregnant females; Stincic et al., 2018), species (rats vs mice; Wittmann et al., 2013), or the use of RNAscope, which amplifies the mRNA signal.

We next investigated other neuronal AT1aR-containing phenotypes that might act locally to stimulate POMC neurons. AngII (Steele, 1992) and kisspeptin (Han et al., 2015) can each stimulate luteinizing hormone (LH) secretion. Moreover, ArcN kisspeptin neurons, via release of glutamate, can activate POMC neurons (Qiu et al., 2018). Therefore, we determined whether kisspeptin neurons express the AT1aR. However, AT1aR did not colocalize with kisspeptin, although ∼15 ± 3% of AT1aR-positive cells were within 25 μm of kisspeptin neurons (data not shown).

**Figure 18.**
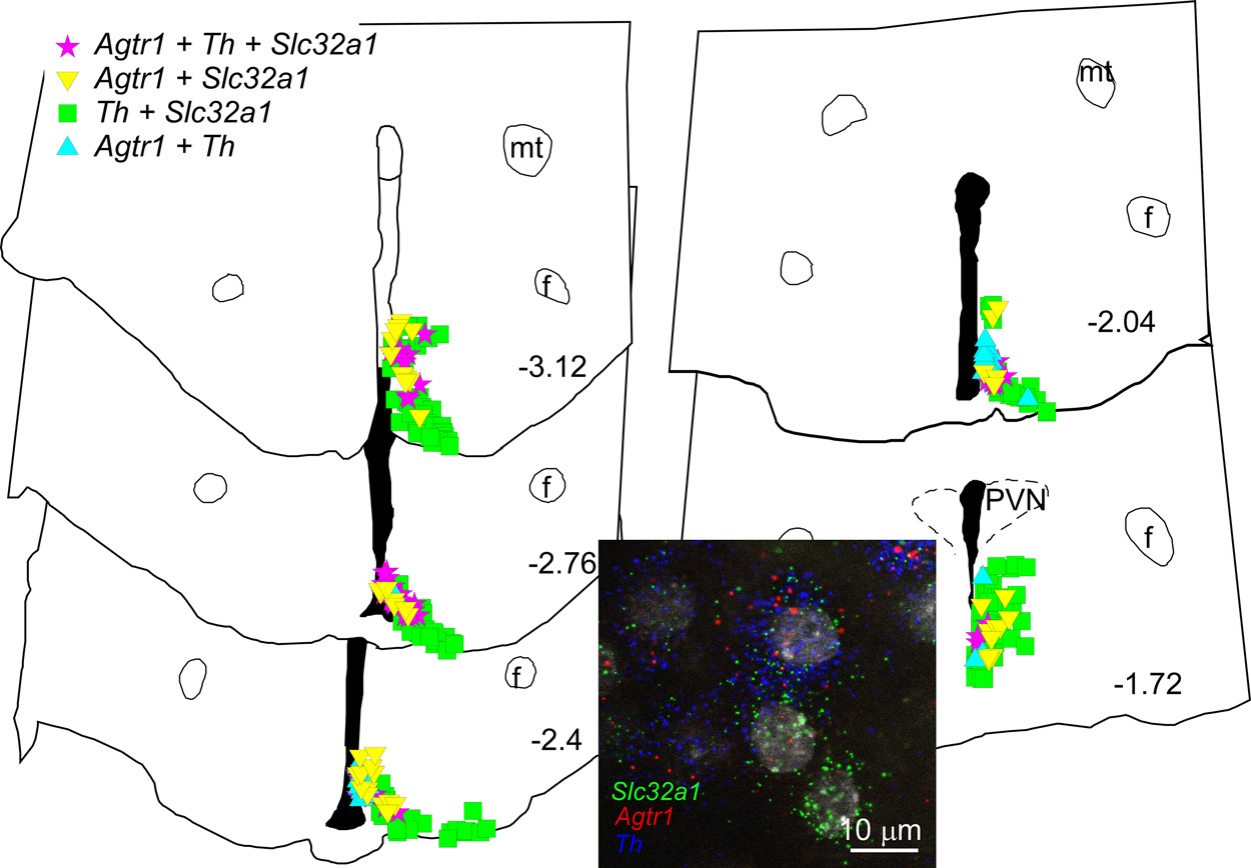
AT1aR (*Agtr1*) are expressed in GABAergic (*Slc32a1*) TH (*Th*) neurons. Computer stage-assisted drawings of hypothalamic coronal sections through the ArcN showing the distribution of *Agtr1 + Slc32a1+Th* (magenta stars) amid *Agtr1 + Slc32a1* (yellow inverted triangles), *Agtr1 + Th* (blue triangles) and *Th + Slc32a1* neurons (green squares). Approximate millimeters behind bregma (after Paxinos and Watson, 2013) indicated by numbers in lower right of each section. Abbreviations as in Figures 16, 17. Inset, Photomicrograph of RNAscope assay for *Agtr1*, *Slc32a1*, and *Th* in ArcN. *Agtr1* in red, *Slc32a1* in green, and *Th* in blue. Scale bar: 10 μm.

ArcN tyrosine hydroxylase (TH) neurons can release dopamine, and AngII inhibits prolactin secretion via increased dopamine release (Steele, 1992). Further, ArcN TH neurons were shown to express AT1aR in estrogen+progesterone-treated female rats (Jöhren et al., 1997), to mimic estrus or pregnancy. Here, we confirm that many AT1aR neurons co-express TH (68 ± 1%, *n* = 3, Fig. 18); conversely, a significant number of TH neurons expressed the AT1aR (32 ± 4%). Moreover, and as shown previously (Zhang and van den Pol, 2015; Marshall et al., 2017), most TH neurons, like AT1aR neurons, were GABAergic (express VGat, 84 ± 5%).

## Discussion

While previous work indicated that systemic AngII activates the ArcN (Davern and Head, 2007), Sapru and colleagues were the first to demonstrate that ArcN AngII increases AP (Arakawa et al., 2011). Here, we show that the pressor response exhibits two phases, both of which are mediated by activation of AT1aR: an initial rapid phase, particularly prominent in females, is mediated by vasopressin-induced vasoconstriction, and the second phase evident in both sexes is associated with slowly developing increases in LSNA, SSNA, and HR. In females, we further show that the effects of ArcN AngII vary during the estrous cycle, with significant increases in LSNA, SSNA, HR, and MAP occurring during diestrus and estrus, but only a pressor response during proestrus, and that pregnancy markedly increases the expression of AT1aR in the ArcN with parallel substantial AngII-induced increases in SNA and MAP. In both sexes, the sympathoexcitation relied on suppression of tonic sympathoinhibitory NPY inputs, and activation of POMC/α-MSH projections, to the PVN; DMH Y1R were not involved. Our finding that few or no NPY or POMC neurons express the AT1aR suggests that AngII elicits these effects at least in part indirectly via local interneurons. However, the lack of co-expression with kisspeptin eliminated this neuronal type as a candidate. Instead, AT1aRs were found in TH (presumed dopaminergic) neurons that are largely GABAergic. Collectively, these data suggest that ArcN AngII increases SNA and AP at least in part via TH interneurons, resulting in suppression of tonic NPY sympathoinhibitory, and stimulation of POMC sympathoexcitatory, projections to the PVN.

While a role for the ArcN in cardiovascular control is well accepted, the present results are the first to show that the ArcN-AngII-induced pressor response is mediated in part by sympathoexcitation; more specifically, bilateral (but not unilateral) ArcN AngII activation of AT1aR produced a slowly developing and sustained increase in the activity of sympathetic nerves innervating the hindlimb and the splanchnic circulation, which implicates engagement of ArcN cellular signaling mechanisms. These responses were observed in both males and females, although estrous and pregnant females exhibited the greatest increases in AP and SNA, in parallel with increased ArcN AT1aR expression. In both sexes, prior injections of AngII into the ArcN prevented the sympathoexcitatory response normally induced by blockade of PVN NPY Y1R. The failure of PVN BIBO3304 to increase SNA after ArcN AngII injections is not because of a ceiling effect, since DMH BIBO3304 triggered a further normal increase in SNA after ArcN AngII, and because other agonists that increase SNA via NPY/POMC projections to the PVN, like insulin (Cassaglia et al., 2011, 2016; Ward et al., 2011), can produce even greater increases in SNA. Therefore, we conclude that tonically inhibitory NPY inputs to the PVN were silenced by ArcN AngII. This conclusion is consistent with a previous study in mice showing that genetic deletion of AT1aR from ArcN AgRP neurons increased NPY expression within the ArcN (Morselli et al., 2018). ArcN AngII also recruits sympathoexcitatory POMC inputs into the PVN, since SHU9119 decreased SNA after ArcN AngII in both sexes. More importantly, prior PVN SHU9119 pretreatment completely prevented the sympathoexcitatory effects of ArcN AngII in males, indicating that ArcN POMC neurons that project to the PVN are a major component of the sympathoexcitatory response to ArcN AngII. The synergism between the decreases in NPY and increases in POMC inputs into the PVN is consistent with prior studies showing that all PVN presympathetic neurons that are inhibited by NPY are activated by α-MSH (Cassaglia et al., 2014), that stimulation of PVN presympathetic neurons by α-MSH requires simultaneous withdrawal of tonic NPY inhibition (Shi et al., 2015b), that blockade of PVN MC3/4R with SHU9119 prevents the increase in SNA induced by PVN BIBO3304 (Cassaglia et al., 2014), and that experimental or physiological states that increase SNA via the ArcN, like leptin (Shi et al., 2015b) or insulin (Ward et al., 2011; Cassaglia et al., 2016) administration, pregnancy (Shi et al., 2015a), or obesity (Shi et al., 2019a, 2020b), are all mediated by decreased PVN NPY Y1R and increased PVN MC3/4R activity. On the other hand, while ArcN NPY neurons via Y1R (Shi et al., 2017; and likely also POMC neurons) are capable of influencing SNA via an action in the DMH, our data do not support a role for this linkage in the sympathoexcitatory effects of ArcN AngII.

Using RNAscope, AT1aRs were found throughout the ArcN, although in males and diestrus or proestrus females, at much lower levels than in nearby hypothalamic nuclei, like the DMH or VMH. However, only a small fraction (∼10%) of AT1aRs were expressed in NPY neurons in both males and females, and a scattered few (males) or no (females) AT1aRs were found in POMC neurons. These findings raise several important questions. First, how can ArcN AngII increase SNA in male and diestrous rats, if receptor expression is low? Based on the slowly-developing nature of the response, and the failure of candesartan to reverse the response, signaling mechanisms may be engaged that amplify the initial signal, as is typical of G-protein coupling. In addition, ArcN AngII may increase the expression of its own receptor, as in other hypothalamic areas (Xue et al., 2012). In this context, it is notable that leptin can also induce the expression of its own receptor (Shi et al., 2020a) and that obesity and leptin (Hilzendeger et al., 2012), and in females progesterone (Jöhren et al., 1997; Donadio et al., 2006), can increase AT1aR expression.

The present results suggest two mechanisms by which AngII could inhibit NPY neurons. First, since AT1aR do co-localize with some NPY neurons, AngII may directly hyperpolarize or inhibit this cohort, although to our knowledge AT1aR-mediated neuronal inhibition has not been reported previously. Second, as the majority of AT1aR neurons also express VGat, and are often nearby NPY neurons, AngII-AT1aR-mediated stimulation of GABAergic neurons could locally inhibit nearby NPY neurons. On the other hand, the paucity of AT1aR expression in POMC neurons raises a second important question: how are POMC neurons activated to drive the sympathoexcitatory response? Multiple mechanisms could be involved. First, AngII-induced loss of tonic NPY sympathoinhibition within the PVN could unveil unfettered tonic POMC sympathoexcitation. Second, ArcN POMC presympathetic neurons, which are likely a small component of the entire POMC population, may be among the few POMC neurons that express AT1aR (only in males). In support, in obese males (not females), POMC presympathetic neurons become sensitized to the sympathoexcitatory effects of leptin and insulin (Shi et al., 2019a, 2020b); however, simultaneously, most ArcN POMC neurons of obese males are resistant to the anorectic effects of leptin and insulin (Prior et al., 2010; Mark, 2013). Third, AT1aR-induced (direct or indirect) hyperpolarization of NPY neurons might release neighboring POMC neurons from tonic NPY inhibition (Roseberry et al., 2004; Atasoy et al., 2012), thereby increasing their activity. Finally, AngII could excite ArcN AT1aR interneurons, which in turn activate POMC neurons. Kisspeptin neurons were considered a strong candidate, since kisspeptin neurons can stimulate POMC neurons via release of glutamate (Qiu et al., 2018). However, glutamatergic AT1aR neurons were relatively few and rarely nearby POMC neurons. More importantly, co-expression of kisspeptin and AT1aR was never observed.

As previously noted in sex-steroid-treated female rats (Jöhren et al., 1997), we found instead that a significant fraction of AT1aR-positve cells also expressed TH. Most TH and AT1aR-expressing cells were localized within the dorsomedial (dm) ArcN, and previous studies in rats (Zoli et al., 1993) and mice (Zhang and van den Pol, 2015) revealed that TH neurons in the dm ArcN express and release dopamine, rather than norepinephrine or epinephrine, in addition to GABA (confirmed here). ArcN TH neurons send axons locally (Zhang and van den Pol, 2015, 2016) and inhibit a large fraction of nearby neurons (both TH and non-TH) via GABA release (Zhang and van den Pol, 2015). Yet, in 11 cases in mice, no electrophysiologically apparent synaptic connection between ArcN TH neurons and identified NPY neurons was observed (Zhang and van den Pol, 2016). Therefore, if AT1aR-TH neurons that release GABA inhibit NPY neurons, this must occur via a subset of TH-AT1aR neurons with direct connections to NPY neurons or via bulk diffusion of GABA to extrasynaptic sites (Belelli et al., 2009; Lee and Maguire, 2014). Collectively, current information suggests that ArcN AngII increases SNA via release of α-MSH in the PVN from ArcN POMC neurons, by disinhibition of NPY neurons both in the PVN and likely also the ArcN.

Pregnancy slowly increases basal SNA, to reach very high levels just before delivery (Brooks et al., 2020). However, the mechanism is unknown. One candidate is central AngII actions. Indirect support includes the findings that pregnancy increases plasma AngII levels in parallel with the increases in SNA, that the increase in muscle SNA in women correlates with the increase in renin, that the pressor response to intracerebroventricular AngII is larger in pregnant compared with nonpregnant rats due in part to greater activation of the sympathetic nervous system, and that intracerebroventricular administration of losartan, an AT1aR antagonist, decreases RSNA (relative to MAP) in late pregnant conscious rabbits (for review, see Brooks et al., 2020). However, the central sites at which AngII binds to AT1aR to support elevated SNA have not been identified. The present results suggest that the ArcN may be one candidate, since pregnancy markedly increased ArcN AT1aR expression. In addition, pregnancy enhanced the sympathoexcitatory and pressor responses to ArcN AngII, at least compared with proestrus, another reproductive state with high gonadal hormone levels. However, proof of this hypothesis requires evidence that blockade of ArcN AT1aR decreases SNA in late pregnant individuals.

AngII was originally shown to stimulate vasopressin secretion 50 years ago (Bonjour and Malvin, 1970) by acting centrally (Mouw et al., 1971). Since these initial observations, a large body of work indicates that AngII binds to AT1R in circumventricular organs (Brooks and Malvin, 1993; McKinley et al., 2004) as well as hypothalamic sites behind the blood-brain barrier, such as the PVN and supraoptic nucleus (Prager-Khoutorsky and Bourque, 2010) to enhance vasopressin release. The present results reveal a new site of action for AngII to stimulate vasopressin, the ArcN, since blockade of systemic vasopressin type 1 receptors prevented the initial pressor response to ArcN AngII nanoinjections in nonpregnant rats and completely prevented the AP rise during pregnancy. Our data do not explain the mechanisms by which ArcN AngII stimulates vasopressin secretion, but there are many possibilities. First, AT1aR-expressing neurons in the PVN project to the inner zone of the median eminence (ME; de Kloet et al., 2017; where vasopressin magnocellular neurons travel to the posterior pituitary and can be activated; Holmes et al., 1986); therefore, in parallel, ArcN-AT1aR activation may stimulate vasopressin magnocellular vasopressin neurons in passage in the ME. Indeed, it is well established that ArcN DA neurons project to the ME to inhibit prolactin secretion and that that the majority of ArcN TH (DA) neurons express the AT1aR (Jöhren et al., 1997; Fig. 18). Moreover, the ME and posterior pituitary express excitatory D1 receptors and are innervated by DA neurons (Björklund et al., 1973; Huang et al., 1992), and ArcN DA stimulates vasopressin secretion (Gerstberger et al., 1987; Rossi, 1998; Gálfi et al., 2001). Thus, it is tempting to speculate that ArcN AngII stimulates posterior pituitary vasopressin secretion via DA-D1 receptor stimulation in the ME and/or posterior pituitary. Alternatively, the rapidity of the response implicates the actions of a fast neurotransmitter, like glutamate, dopamine, or GABA, possibly via ArcN projections to magnocellular neurons in the PVN or SON. Prior studies revealed that ArcN neurons that express neurokinin B/kisspeptin, and are largely glutamatergic, project to and activate vasopressin neurons in the SON and PVN (Pineda et al., 2016; Stincic et al., 2021). However, here we show that, at least in the rat, kisspeptin neurons do not express the AT1aR. On the other hand, ArcN TH neurons project to the PVN (Zhang and van den Pol, 2016), and PVN magnocellular neurons express excitatory D1 receptors (Ran et al., 2019). Thus, AngII-induced excitation of ArcN TH neurons could stimulate vasopressin secretion via activation of D1 receptors in the PVN. Future research is required to test these and other possible hypotheses to identify the mechanisms by which ArcN AngII stimulates vasopressin release.

Pregnancy increases vasopressin secretion, such that the relationship between plasma vasopressin levels and osmolality is left-shifted, producing frank hyponatremia/decreased plasma osmolality. Indeed, in the present study, iv injection of the vasopressin antagonist lowered AP in anesthetized, acutely prepared pregnant, but not virgin, rats, indirectly suggesting relatively elevated vasopressin levels during pregnancy. Current evidence suggests that the relative increase in vasopressin is mediated by relaxin, which synergizes with AngII, in the lamina terminalis (for review, see McKinley et al., 2004; Brunton et al., 2008; Brooks et al., 2020). Our finding that ArcN AngII likely elicits enhanced vasopressin secretion during pregnancy identifies the ArcN as a potentially additional site at which AngII stimulates vasopressin secretion in pregnant animals.

**Figure 19.**
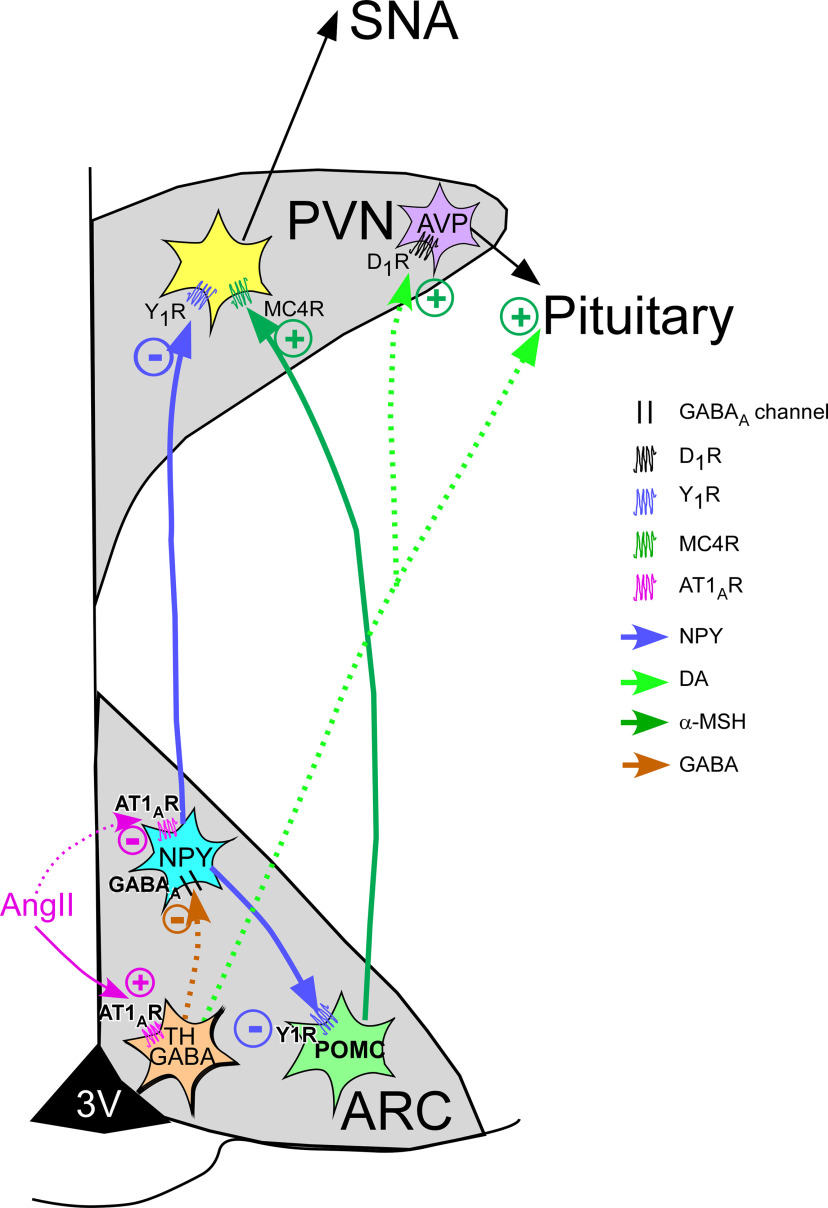
Hypothetical model summarizing the results and conclusions. AngII binds to AT1aR and stimulates TH GABAergic interneurons, which suppress NPY neuronal activity. Alternatively, AngII binding to AT1aR may directly inhibit NPY neurons. NPY neurons tonically inhibit PVN preautonomic neurons. Release of this NPY inhibition allows ArcN POMC neuronal activity to activate PVN MC4R on presympathetic neurons, by both disinhibition of POMC neurons in the ArcN and also by unfettered activation of PVN presympathetic neurons by α-MSH. As a result, SNA increases. Simultaneously, AT1aR activation of ArcN TH neurons that project to the PVN, ME, or pituitary may release DA to stimulate vasopressin secretion via D1 receptors. Solid arrows indicate known functional connectivity. Dotted arrows require further experimentation to establish.

Collectively, these data suggest the following functional model by which ArcN AngII increases SNA and BP (Fig. 19): AngII binding to AT1aR directly inhibits ArcN NPY neurons and/or stimulates TH GABAergic interneurons, which suppress NPY neuronal activity. ArcN NPY neurons tonically inhibit PVN preautonomic neurons. Release of this NPY inhibition allows ArcN POMC neuronal activity to activate PVN MC4R on presympathetic neurons, by both disinhibition of POMC neurons in the ArcN and also by unfettered activation of PVN presympathetic neurons by α-MSH. We hypothesize that simultaneously, particularly in females, AT1aR activation of ArcN TH neurons that project to the ME, pituitary, or PVN release DA to stimulate vasopressin secretion.

The ArcN is a key integrative site in the control of reproduction and energy balance, which in turn are influenced by ArcN AngII-AT1aR (Steele, 1992; Donadio et al., 2006; Deng and Grobe, 2019). The present results further demonstrate that ArcN AngII actions at AT1aR increases AP through stimulation of SNA via projections to the PVN and also via vasopressin secretion. Since the original discovery by Vander and colleagues that psychosocial stress stimulates renin secretion (Clamage et al., 1976), it has become increasingly clear that central activation of AT1aR contributes to a multitude of both physical and psychological stress responses, including increases in vasopressin secretion and activation of the sympathetic nervous system (for review, see Saavedra et al., 2005, 2011; Mayorov, 2011). Thus, ArcN AT1aR are well-poised to facilitate integration of these modalities with stress. Indeed, ArcN AT1aR are required for stress to inhibit prolactin secretion (Donadio et al., 2004). Moreover, because brain TH neurons, through the release of norepinephrine and DA, also mediate multiple stress responses (Anisman and Zacharko, 1986), the association of AT1aR with TH in neurons that project within and outside the ArcN further points to a local/regional integrative role with stress. This local role could be similar to that recently described for AT1aR-CRF crosstalk within the PVN to control both the HPA axis and autonomic control of AP in the context of stress (de Kloet et al., 2017; Elsaafien et al., 2021).

The initial cardiovascular event during pregnancy is profound vasodilation, which tends to lower AP and activate the RAS (Brooks et al., 2020), and as such presents a physical stress. Intriguingly, in females, ArcN AT1aR expression is dramatically increased by high progesterone in association with estrogen, such as shown here during pregnancy, as well as during estrus (Jöhren et al., 1997). Thus, as described above, increased actions of ArcN AT1aR (because of both increased AT1aR and AngII) may contribute to ArcN support of elevated SNA and BP during pregnancy (Shi et al., 2015a). The elevated AT1aR may also suppress prolactin secretion (Steele, 1992), until just before delivery when progesterone levels plunge and prolactin levels rise in preparation for delivery and lactation, when brain AT1aR are low (Speth et al., 1999). Clearly, a direct testing of such an ArcN AT1aR-TH integrative role with stress and/or pregnancy awaits further research.

## Synthesis

Reviewing Editor: Christopher Colwell, UCLA School of Medicine Decisions are customarily a result of the Reviewing Editor and the peer reviewers coming together and discussing their recommendations until a consensus is reached. When revisions are invited, a fact-based synthesis statement explaining their decision and outlining what is needed to prepare a revision will be listed below.

In this submission titled “Arcuate Angiotensin II increases arterial pressure via coordinated increases in sympathetic nerve activity and vasopressin secretion in male and female rats: evidence supporting a local integrative role for arcuate AT1aR,” authors utilize an array of methods to explore the role of the RAS within the arcuate nucleus of the hypothalamus in neuroendocrine and autonomic control of blood pressure. Overall the manuscript represents a very large volume of work and provides exciting new data dissecting the cellular biology, anatomy, and physiological significance of angiotensin signalling within the arcuate nucleus for cardiovascular control.

The manuscript is well written and easy to read. Still the reviewers have identified a number of issues that need to be addressed.

Moderate concerns:

The abstract should present clear conclusion(s) drawn solely based on the results of this study, and the number of figures should be reduced. Consider changing the title as this study does not provide evidence supporting a local “integrative” role for arcuate AR1aR.

The possible role of vasopressin in the ArcN AngII-induced increases in ABP and SNA has been studied only in female rats in estrus and late pregnancy, but not in male rats. However, the authors keep stating the role of vasopressin in the male rat throughout the manuscript. This needs to be clarified.

No physiological studies has been conducted to directly support the argument of the authors with respect to the neural circuitry involved in the action of AngII in the ArcN. The results obtained with RNAScope fluorescent in situ hybridization and the related discussion should be reduced accordingly.

Reviewer 1:

Line 72 - Claflin did not assess NPY localization per se, but rather, AgRP colocalization with AT1aR; therefore, suggest adding a sentence somewhere in the introduction explaining that NPY and AgRP strongly colocalize within the ArcN, so that this sentence/interpretation is better justified. Morselli (PMC5882211) also colocalized AT1aR with AgRP in the ArcN of mice, and consistent with the current manuscript’s studies in rats (especially Fig 2), found that genetic deletion of AT1aR from AgRP neurons in mice caused disinhibition of NPY expression within the ArcN.

Fig 3 - The interpretation of these data are confusing. First, the effect size (e.g. MAP increases in the white squares >5 mmHg with DMH BIBO injection even though it was not statistically significant) seems to be at least as large - maybe larger - than the effect of PVN BIBO (i.e. Fig 2 white squares increase maybe 4 mmHg?). Thus, NPY signaling seems to be important for control of these endpoints in both brain regions. Then, ArcN AngII injection stimulates the various endpoints, and this seems to be additive / synergistic with DMH BIBO (Fig 3). The authors conclude that (Lines 212-213) “... NPY projections to the DMH are not involved in the sympathoexcitatory effects of ArcN AngII.” It could be argued that this experimental design does not really test that conclusion, and that an NPY receptor agonist (or NPY itself) should be used to test whether the effects of AngII on DMH NPY projections are involved in sympathoexcitatory control. Suggest rewording this conclusion or repeating experiment with NPY injection into the DMH, to determine whether ‘loss of NPY signaling to the DMH’ is required for AngII control of these endpoints. To these ends, a similar argument could be made for the data in Fig 2; the lack of additive effect of PVN BIBO after ArcN AngII injection could mean that ArcN AngII works via PVN NPY signaling, or it could equally well mean that the injection pipette was clogged, or that the AngII-activated mechanism is separate from and dominant over the NPY-mediated mechanism. Although these experiments are consistent with the conclusions that the authors propose, they do not directly test those conclusions either. Rewording the conclusions here, or addition of experiments with NPY agonism, would bolster this part of the manuscript. (This same conclusion is referenced and should probably be softened on lines 224-226.)

Is it possible that other, BIBO-insensitive, NPY receptors mediate effects of ArcN AngII-stimulated NPY projections to other brain regions outside the PVN (such as the DMH)?

Data in Figure 5 are exciting and clear, but the addition of a control such as an injection of SHU9119 into a different brain region to demonstrate that the SHU9119 compound’s action ‘within the PVN’ is specific would be really outstanding.

With regard to Fig 6 data, the authors might consider adding some discussion of the possible impact of AngII delivery to the ventromedial ArcN versus dorsal/lateral regions of the ArcN. This is briefly touched in the current discussion, but perhaps the authors have more thoughts to mention.

Throughout, many (not all) of the panels need minor correction: Y-axes should be labelled as “delta” for each endpoint (e.g. “delta MAP,” not simply “MAP”). Along with this issue, were there any baseline differences in the male rats? The authors do a nice job demonstrating similar baseline values for females in Table 2, but such data are not obvious for males.

The implication of AVP type 1 receptors in the MAP responses of the estrous and pregnant rats to ArcN AngII is fascinating. Interpretation of the SNA responses in pregnant rats after AVP1x vs saline are complicated, however, as the data are presented as % control (i.e. baseline within that animal?). Is the baseline activity of LSNA and/or SSNA higher in pregnant rats, such that the increase at 90 min after injection is reaching the same maximum (in absolute units)?

Along these lines, it is well known that AVP secretion increases in early gestation, and defends a lower plasma osmolality and expanded vascular volume; do baseline differences in AVP secretion explain the difference in effects of AVP1x (itself, alone) upon MAP? Does ArcN AngII contribute to osmoregulation through projections to the SON and/or PVN (what happened, if anything, to plasma osmolality after ArcN AngII injection)? In other words, does ArcN AngII directly stimulate AVP release by the SON or PVN, or does ArcN AngII action change osmosensitivity of magnocellular AVP neurons?

The line graph in Fig 15 is oddly labelled; Do the authors mean “percent of NPY & POMC neurons expressing AT1aR” at each level relative to bregma? Or is this a measure of relative AT1aR transcript density (i.e. “percent of total AT1aR across these sections combined, as distributed across each level”)? It seems more likely to be the former, but a more straightforward Y-axis label would be helpful.

In Fig 15, the authors show example NPY cells that have strong expression of NPY (as expected for a transcript encoding a secreted peptide product), and a few dots indicating AT1aR expression. This low expression of AT1aR also makes sense, as AT1aR is a GPCR and expected to be expressed at low levels. In contrast, in the neighboring panel illustrating POMC neurons, the example cell that is blown up to illustrate colocalization shows only a few dots of “POMC.” POMC is the transcript for a secreted protein, and therefore (as obvious in many other cells in the larger image) typically expressed at very high levels in the cell. There is concern that the example cell is perhaps not a canonical POMC neuron. Is AT1aR expressed in any of the cells with strong expression of POMC? Is this some other cell type that exhibits very low expression of POMC (for example, there are many, many dissociable subtypes of cells within the ArcN that express POMC according to Romanov 2017, Campbell 2017, and other single cell RNAseq experiments)? Or is this just “noise” in the assay? Some kind of qualitative or semi-quantitative regression between “level of AT1aR expression vs intensity of POMC expression” might help clarify this issue. The conclusion (line 310) “... rarely” is appropriate, but maybe a little more discussion is justified here.

The authors are cautioned not to over-interpret the biological significance of expression levels of mRNA’s encoding GPCR’s (e.g. lines 410-412). The protein levels, binding kinetics / cell surface localization, and second-messenger signal coupling efficiency of AT1aR are very unlikely to be sensitive to relatively small changes in mRNA expression (from “a little” to “a little more”). Without additional data to directly assess the sensitivity of the neurons to AngII in the various states (sexes, estrous phases, etc.) when AT1aR expression varies within a given cell. In contrast, the authors are probably correct to suggest that the gain (from “zero” to “any”) of AT1aR expression in new neurons during pregnancy likely has physiological consequences.

Line 470-471, etc. - Recent studies by Sandgren et al. and by Frazier et al. (e.g. PMC6032302 and PMC7896018) indicate that AT1aR is expressed on AVP neurons of the SON, but not the PVN, which supports a more complicated /multisynaptic architecture of AngII-controlled AVP-produced circuits within the PVN. Given the current study’s implication of ArcN AngII action to stimulate AVP for BP control, perhaps this new insight will help resolve this relationship inside the PVN? Further, there is evidence that magnocellular neurons in the SON express the melanocortin MC4R, which may also mediate some of this control.

Reviewer 2:

1) line 51. Define MC3/4R

2) line 198-201. This reviewer cannot follow the authors’argument.

3) line 237. Change the phrase “rat during proestrus and diestrus” to “proestrus and diestrus rats"

4) line 257. Replace the word “indicate” by “suggest"
